# Arbitrary Orthogonal Polarization Decomposition and Routing With Complex Amplitude Modulation via Wheel‐of‐Fortune‐Inspired Metasurfaces

**DOI:** 10.1002/advs.202600046

**Published:** 2026-03-17

**Authors:** Tong Liu, Changhong Dai, Weike Feng, Yanzhao Wang, Wenxin Liu, Wentao Zhang, Chao Tian, Guangwei Hu, He‐Xiu Xu

**Affiliations:** ^1^ Air and Missile Defense College Air Force Engineering University Xi'an China; ^2^ School of Electrical and Electronic Engineering Nanyang Technological University Singapore Singapore

**Keywords:** amplitude‐phase, arbitrary polarization, router, transmitting and receiving patches

## Abstract

Multi‐dimensional (amplitude, phase, and polarization) manipulation of electromagnetic (EM) waves has long been a central goal in optics. However, most reported metasurface designs to date have achieved only one‐ or two‐dimensional manipulation due to losslessness and in‐plane symmetry constraints. To address this challenge, we propose and experimentally demonstrate a receiver‐transmitter‐integrated metasurface consisting of receiver, transmitter, and connector inspired by the prize‐selection mechanism of “Wheel‐of‐Fortune”. It functions as a full polarizer, decomposing an incident wave into two orthogonal components and routing them separately into transmission and reflection channels, while enabling three‐dimensional control of transmitted component. To engineer this, we establish a *fundamental theory* to tailor in‐plane symmetries of transmitting and receiving patches for polarization‐routing transmission and reflection through both global and local rotations of these resonators. Specifically, the amplitude and phase of the transmitted component are set by parametric tuning of C‐slot resonators of the receiver and transmitter, and stripline lengths of the connector, respectively. We finally realize and experimentally demonstrate two metadevices that achieve polarization‐routing transmissive‐reflective holography and an asymmetric beamformer with unequal power and deflection angle, respectively. This work provides a powerful platform to realize multidimensional control of EM waves, which can inspire numerous future applications for next‐generation devices.

## Graphical abstract

This work demonstrates a receiver‐transmitter‐integrated metasurface that decomposes an incident wave into orthogonal components and routes them into separate channels. Inspired by a “Wheel‐of‐Fortune” mechanism, it enables independent control over the amplitude, phase, and polarization of the transmitted wave. The platform is experimentally validated through polarization‐routing holography and an asymmetric beamformer.

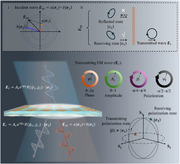

## 1 Introduction

Independent control of electromagnetic (EM) wave properties, including polarization, amplitude, and phase, is critical for advanced devices spanning microwave to optical frequencies [1, 2, 3, 4]. For instance, precise amplitude and/or phase manipulation are essential for imaging systems [5] and optical communication [6, 7, 8, 9, 10], while independent control of polarization and its spatial routing into distinct channels plays a crucial role in applications in data encryption, optical storage, and vectorial information processing [11, 12, 13, 14, 15]. However, traditional devices [16, 17] typically rely on bulky assemblies: achieving amplitude, phase, and/or polarization control often needs complex combinations of discrete components, suffering from restrictions on large size, high complexity, and low efficiency.

Metasurfaces, the planar counterparts of subwavelength metamaterials, have emerged as ultra‐compact and multifunctional platforms for EM waves manipulation. A broad spectrum of effects has been demonstrated, such as wavefront shaping [18, 19, 20, 21], polarization control [22], vectorial field modulation [23, 24, 25], and metasurface cloak [26]. Particularly, single meta‐atoms combining both propagation phase [27, 28] and geometric phase [29] have been employed to realize either the independent phase‐polarization control or simultaneous amplitude‐phase modulation for specific polarization components [30]. These mechanisms further facilitate spin‐decoupled phase manipulation [19, 20, 31, 32] or amplitude modulation [33, 34] for arbitrary pairs of orthogonal polarization states. Despite these advances, the ability to decompose and route arbitrary orthogonal polarization components remains rare. Achieving independent control of the amplitude, phase, and polarization of the routed wave is particularly challenging due to mirror symmetry and the unitary Jones‐matrix constraint [20, 30, 35, 36, 37]. Conventional approaches to polarization routing often suffer from limited EM dimensional control, polarization states [38], and constrained efficiency. They cannot typically independently modulate the full set of wave properties (amplitude, phase, and polarization) for the routed component after decomposition.

In this study, we propose a receiver‐transmitter‐integrated metasurface functioning as a full polarizer. Its core operation is threefold: decomposing an arbitrary incident wave into two predesigned orthogonal polarization components, routing them separately into transmission and reflection space, and enabling independent control of amplitude, phase, and polarization of transmitted components. Each meta‐atom consists of receiving and transmitting patches (a C‐slot, bisymmetric split‐ring resonators), and a connector part that incorporates two metallized vias and a stripline. In our design, the polarization control mechanism operates in a manner analogous to a “Wheel of Fortune.” The C‐slot and bisymmetric split‐ring resonators function as a “pointer” and “prize disc,” respectively, and their synergistic interaction thereby defines the resultant polarization state (the “prize”) of the target electromagnetic wave. By exploiting the relationship between polarization state and the relative rotation of resonators, we realize polarization‐routing and manipulation of incident waves. Moreover, by tuning the C‐slot resonators’ parameters and the stripline length, amplitude (0–1) and phase (0–2π) for arbitrary polarization state are independently modulated. Consequently, the metasurface realizes orthogonal polarization decomposition and routing with complex amplitude and polarization modulation, thus operating as a full polarizer that generates a pre‐determined output polarization independent of the incident polarization state. Therefore, our integrated capability marks two distinct advantages from available metasurfaces with two‐ensional or three‐dimensional (amplitude, phase, and polarization) manipulation of EM wave [36, 37], which were limited by spin‐locked or symmetry‐restricted mechanisms, respectively (see Figure S1 and Table S1 for detailed comparison). Specifically, these advantages are mainly manifested in two key aspects: a functionally decoupled architecture that allows independent wavefront control (with full control over the amplitude, phase, and polarization [36] of the transmitted wave) without perturbing the polarization‐routing function; and a receiving layer that operates independently of incident polarization state (linear, circular [37], or elliptical), granting universal applicability. Our work establishes a new paradigm for resolving the longstanding challenge of simultaneous, independent, and high‐efficiency multi‐dimensional EM wave manipulation.

## 2 Results and Discussion

### 2.1 Concept and Fundamental Theory

**FIGURE 1:**
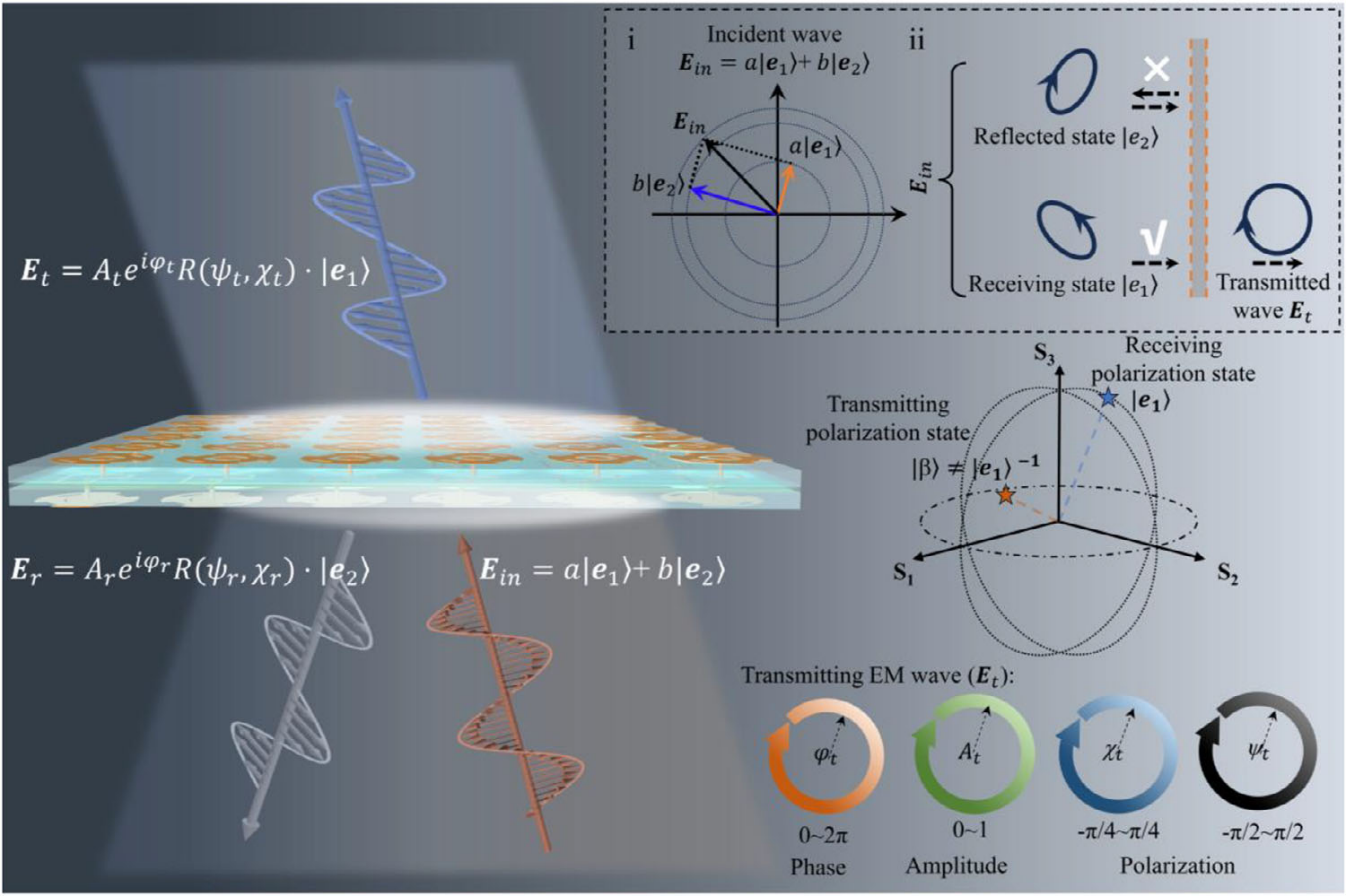
Schematic of the Wheel‐of‐Fortune‐inspired metasurface and performance for arbitrary, independent control of amplitude, phase, and polarization. The receiving and transmitting polarization states are denoted by $|e_{1}\rangle =\left(\begin{array}{c} \cos\chi_{i}\\ e^{i2\psi_{i}}\sin\chi_{i} \end{array}\right)$ and $\left|\beta \right\rangle =\left(\begin{array}{c} \cos\chi_{t}e^{i(\zeta_{1}+\zeta_{2})}\\ -\sin\chi_{t}e^{i(\zeta_{2}-\zeta_{1})} \end{array}\right)$, respectively, where *ψ_i_* and *χ_i_* (*ψ_t_* and *χ_t_*) represent the polarization azimuth angle and ellipticity angle of the incident (transmitted) EM wave. The insets (i) and (ii) illustrate the polarization decomposition and routing principle of the EM wave, respectively. Here, the incident wave ***E**_in_* is expressed as a superposition of orthogonal polarization states: ***E**_in_* = *a*| ***e***_1_〉 + *b*| ***e***_2_〉, where | ***e***_1_〉 represents polarization state of the receiving patch, and | ***e***_2_〉 is its orthogonal counterpart. The *a* and *b* correspond to complex coefficients of each polarization component.

To illustrate the arbitrary orthogonal polarization decomposition and routing concept, we employ the Jones matrix formalism to rigorously describe the independent manipulation of amplitude, phase, and polarization enabled by the proposed metasurface. As shown in Figure 1, the metasurface is assumed to be illuminated by a normally incident plane wave with an arbitrary polarization state, denoted by ***E*** *_i_*, and the transmitted and reflected fields are related to the incident field through **E**t = **J**t **E**i, **E**r  = **J**r **E**r, where ***J*** *_t_* and ***J*** *_r_* denote the transmission and reflection Jones matrices of the metasurface, respectively. The central question then becomes: what properties should the metasurface possess to function as a full polarizer with amplitude and phase control under arbitrary incident polarization?

To map an arbitrarily polarized incident state onto the transmission and reflection channels, the proposed receiver‐transmitter‐integrated metasurface should first act as a polarization router, selectively coupling specific polarization states into the corresponding channels. Assuming that the receiving patch is configured to respond to the polarization state | ***e***_1_〉, the incident component aligned with the designated receive polarization $|e_{1}\rangle =\left(\begin{array}{c} \cos\chi_{i}\\ e^{i2\psi_{i}}\sin\chi_{i} \end{array}\right)$ is coupled into the forward channel and re‐radiated, whereas components in the orthogonal state $|e_{2}\rangle =\left(\begin{array}{c} -\sin\chi_{i}\\ e^{i2\psi_{i}}\cos\chi_{i} \end{array}\right)$ are rejected and routed to reflection, yielding near‐zero transmission for that polarization(see inset (ii) of Figure 1). This polarization routing behavior can be naturally described using a projection operation. Defining the projector onto | ***e**_i_* 〉 as ***P**_i_* = | ***e**_i_* 〉〈 ***e**_i_* |, one obtains ***P**_i_* | ***e**_i_* 〉 = | ***e**_i_* 〉 and ***P**_i_* | ***e**_j_* 〉 = 0 for *i*≠*j*. Accordingly, the projecting actions associated with the reflection and transmission channels can be expressed as $P_{r/t}=\lambda_{1}^{r/t}P_{1}+\lambda_{2}^{r/t}P_{2}$, where $\lambda_{1}^{r}=0$, $\lambda_{2}^{r}=1$ for the reflection channel and $\lambda_{1}^{t}=1$, $\lambda_{2}^{t}=0$ for the transmission channel. Accordingly, the reflection and transmission Jones matrix can be factorized as

(1) $$J_{r}=U_{r}P_{2},J_{t}=U_{t}P_{1}$$

where ***U*** *_r_* and ***U*** *_t_* are general 2 × 2 complex matrices responsible for shaping the amplitude, phase, and output polarization of the transmitted and reflected waves, respectively. Given that the incident states can be decomposed as ***E**_in_* = *a*| ***e***_1_〉 + *b*| ***e***_2_〉 (see inset (i) of Figure 1), the reflected and transmitted waves can therefore be written as ***E**_r_* = *b**U**_r_* | ***e***_2_〉 and ***E**_t_* = *a**U**_t_* | ***e***_1_〉. As a result, the metasurface selectively decomposes an arbitrarily polarized incident wave and routes different polarization components into corresponding channels.

Beyond polarization routing, the proposed meta‐atom also functions as a full polarizer with independent amplitude, phase, and polarization control. Each meta‐atom comprises a transmitting patch, a receiving patch, and a connector. To illustrate this capability, we analyze the control of amplitude, phase, and polarization in the circular‐polarization basis. In the circular polarization basis, the router can be designed to convert reflected right‐handed circularly polarized (RCP) to left‐handed circularly polarized (LCP) wave (more details can be found in Section S2), the Jones matrices for reflection and transmission can be expressed as:

(2) $$\begin{array}{c} U_{r}=\left(\begin{array}{cc} 0 & e^{i(t_{2}-2\zeta_{2})}\\ A_{r}e^{i(t_{1}+2\zeta_{2})} & 0 \end{array}\right)\\ U_{t}=A_{t}e^{i\varphi_{t}}\left(\begin{array}{cc} \cos\chi_{t}e^{i\left(\zeta_{1}+\zeta_{2}\right)} & 0\\ -\sin\chi_{t}e^{i\left(\zeta_{2}-\zeta_{1}\right)} & 0 \end{array}\right) \end{array}$$

Here, *χ_t_* is the ellipticity angle corresponding to the amplitude ratio between orthogonal transmitted components, and *φ_t_* denotes the global phase. Concurrently, the parameters *ζ*_1_ and *ζ*_2_ denote the global rotation angles of the transmitting and receiving patches, respectively. In addition, *t_1_* and *t_2_* denote the initial phase differences induced by the geometric structural parameters. For a lossless system, energy conservation requires that the transmission amplitude coefficient *A_t_* and the co‐polarized reflection amplitude coefficient *A_r_* satisfy |*A_r_* |^2^ + |*A_t_* |^2^ = 1. Then, the output EM waves $A_{t}e^{i\varphi_{t}}\left|\beta \right\rangle$ can be written as:

(3) $$U_{t}|e_{1}\rangle =A_{t}e^{i\varphi_{t}}\left(\begin{array}{c} \cos\chi_{t}e^{i\left(\zeta_{1}+\zeta_{2}\right)}\\ -\sin\chi_{t}e^{i\left(\zeta_{2}-\zeta_{1}\right)} \end{array}\right)$$

with the polarization angle *ψ_t_* = *ζ*_1_. These relations establish a universal design paradigm for metasurfaces that enable the three‐dimensional manipulation of transmitted EM waves.

### 2.2 Meta‐Atom Design

**FIGURE 2:**
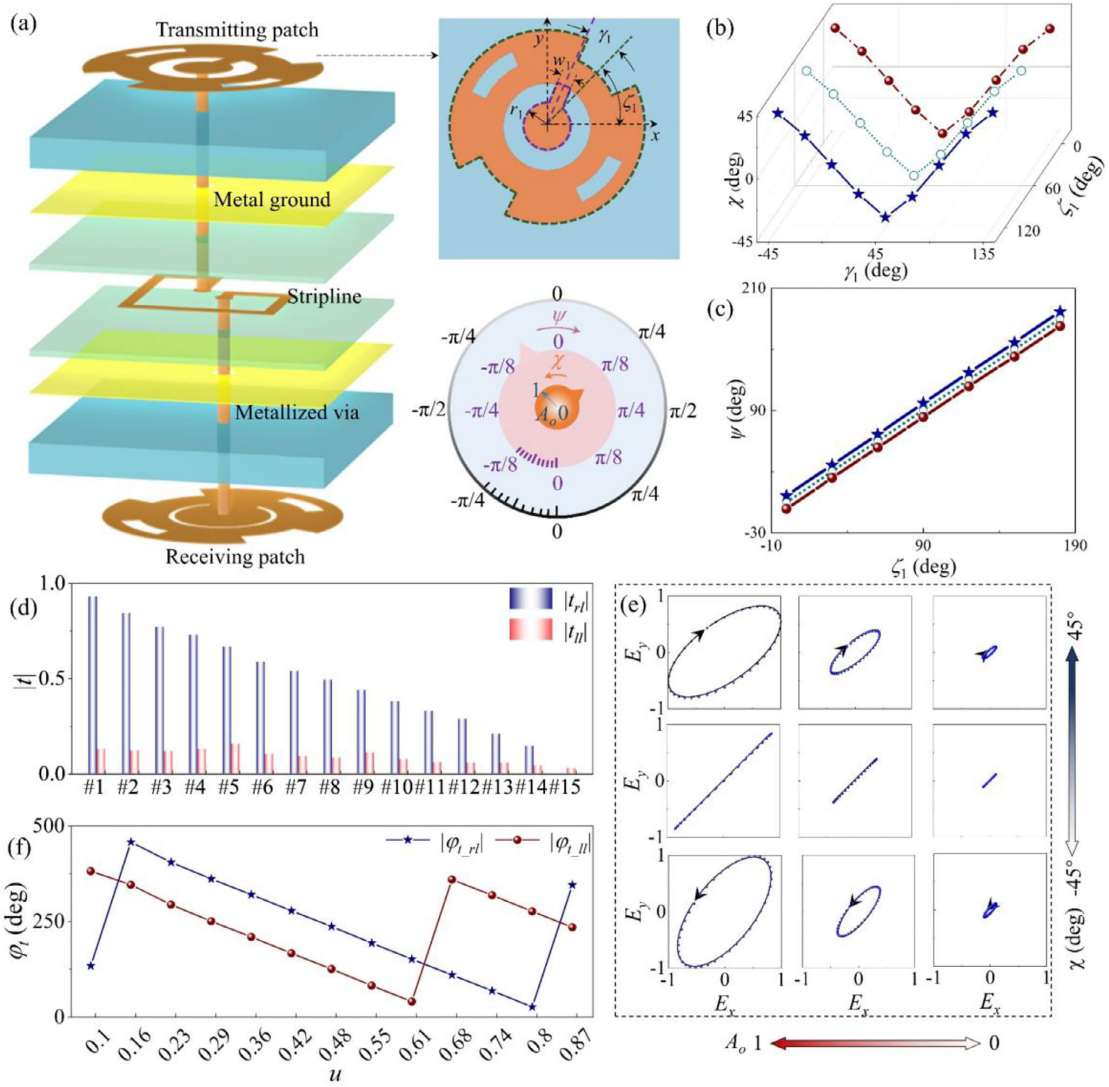
Characterization of the meta‐atom with polarization, amplitude, and phase manipulation. (a) Exploded view of a meta‐atom comprising transmitting and receiving patches, a stripline for output‐phase modulation *φ_t_*, two metallized vias, metal ground, and dielectric substrates (F4bM265 and F4bM350). The transmitting patch features local and global rotation angles (*γ*_1_ and *ζ*_1_) of transmitting patch, with key geometric parameters including inner radius *r*_1_ and gap width *w*_1_ (where *w*_1_ = *r*_1_/*ratio*). The corresponding receiving patch's inner radius and gap width are *r*_7_ and *w*_2_ (*w*_2_ = *r*_7_/3), and the full geometric parameters of the meta‐atom are provided in Figure S2. The metasurface functions as a “Wheel‐of‐Fortune”, enabling continuous tuning of output amplitude *A_o_*, phase *φ_t_*, ellipticity angle *χ_o_*, and polarization angle *ψ_o_*. (b) Ellipticity angle *χ* vs. local rotation angle *γ*_1_ (22.5° step) for *ζ*_1_ = 0°, 60°, and 120°. (c) Azimuth angle *ψ* vs. global rotation angle *ζ*_1_ (30° step) for *γ*_1_ = 22.5°, 0°, and −22.5°. (d) Transmission coefficients (*t_ll_* and *t_rl_*) vs. different parameter combinations of C‐slot resonators of transmitting and receiving patches chosen from the meta‐atom library, with the number of Table S3 as the *x*‐axis. (e) Transmitting polarization states at different transmission amplitudes (*t_rl_* = 0.25, 0.5, and 0.625). (f) Transmission phases vs. different lengths (*u*) parameters of stripline.

We start from establishing a generic design strategy for receiver‐transmitter‐integrated meta‐atoms that function as a polarization router, which allows independent and continuous control of the amplitude, phase, and polarization of the transmitted component. As illustrated in Figure 2a, the proposed receiver‐transmitter‐integrated meta‐atom consists of five metallic layers separated by four dielectric substrates, periodically arranged on a square lattice with a period of λ/3 (λ denotes the operating wavelength, see Figure S2). The meta‐atom integrates a receiving patch, a transmitting patch, and an interconnecting stripline through two metallized vias. When an incident wave illuminates the metasurface, the polarization component aligned with the receiving patch (| ***e***_1_〉) induces a surface current that is captured by the receiving patch and transferred to the transmitting patch through two metallized vias and an interconnecting stripline, which then re‐radiates the transmitted field. In contrast, the orthogonal polarization component (| ***e***_2_〉) is polarization‐mismatched and cannot efficiently excite the resonator, leading to its reflection back into free space. This intrinsic selectivity forms the basis of the polarization‐routing behavior. Both receiving and transmitting patches of meta‐atom adopt a rotational “Wheel‐of‐Fortune” configuration (the right panel of Figure 2a), where the C‐slot resonator acts as a “pointer” and the bisymmetric split‐ring resonator as a “prize disc”. This enables independent control of the polarization of the receiving and transmitting patches (see Figures S3–S5 in Section S3).

Polarization routing manipulation of meta‐atom stems from the in‐plane rotation between the transmitting C‐slot and the bisymmetric split‐ring resonator. The outer ring provides a symmetric background with a fixed polarization response, while the inner C‐shaped slots act as tunable asymmetric elements. By rotating the inner C‐shaped slots, the structure actively breaks the in‐plane symmetry of the patch, inducing a difference in the scattering/transmission amplitudes of the patch for LCP and RCP components and thereby governing polarization eigenstates. Continuous tuning of this amplitude ratio via adjustment of the rotation angle enables dynamic control over the ellipticity angle of the emitted light. Owing to the independent planar symmetries of the receiving and transmitting patches, the meta‐atom supports arbitrary control of both incident and transmitted polarization states.

Specifically, the local relative rotation between the pointer and the disc continuously tunes the ellipticity angle of the transmitted component (*χ* = −π/4–π/4), while the global co‐rotation of both resonators modulates the polarization angle (*ψ* = −π/2–π/2). In the circular‐polarization (CP) basis, *γ*_1_ determines the amplitude ratio *A_RCP_*/*A_LCP_* of transmitted component, thereby the output elliptical angle progresses continuously as γ_1_ increases: from LCP (*γ*_1_ = −45°) state, through left‐handed elliptical states (−45° < *γ*_1_< 0°), to linear polarization (LP, *γ*_1_ = 0°), then to right‐handed elliptical states (0° < *γ*_1_< 45°), and finally to RCP (*γ*_1_ = 45°), as illustrated in Table S2, Figure 2b, and Figures S6–S8. Although *ψ*_1_ slightly shifts with *γ*_1_ (see Figure S9a), this effect can be fully compensated by rotating the entire transmitting patch. Specifically, the global rotation *ζ*_1_ of the transmitting patch imparts a PB phase shift to the orthogonal CP (circularly polarized) components, yielding *ψ* = *ζ*_1_ (see Figure 2c). This phase–polarization relationship holds for all *χ* values, confirming that the polarization and ellipticity angle are independently tunable (see Figure S9b). Under LCP wave incidence, the conversion efficiency, defined as $|E_{T}|=\sqrt{{|T_{ll}|}^{2}+{|T_{rl}|}^{2}}$, exceeds 80.4% for a variety of output polarizations, reaching up to 94.4% for CP outputs (Figure S9c). Further details on the performance under various incident polarizations and γ_1_ values, including the continuous evolution of the output polarization state, are provided in Section S4 (see Figure S10).

The amplitude of the transmitted component (0–1) is primarily determined by the geometric parameters of the C‐slot resonator—namely, its inner radius and gap width. Taking the configuration with an LCP receiver and an RCP transmitter as an example (*γ*_1_ = 45°, *γ*_2_ = −45°, *ζ*_1_ = 0°, *ζ*_2_ = 0°), the transmission coefficient |*t_rl_* | of the designed meta‐atom is continuously tuned from 1 to 0 by adjusting the inner radius (*r*_1_ and *r*_7_ from 3.2 to 0 mm) and gap width (*w*_1_ and *w*_2_ from 1.6 to 0 mm) of the C‐slot resonators in the transmitting and receiving patches under LCP wave illumination (see Table S3 for detailed parameters), while the cross‐polarized transmission coefficients |*t_ll_* | remain quantitatively small and stable throughout the tuning range(Figure 2d). Simultaneously, the reflection coefficient |*r_rl_* | of the meta‐atom varies continuously from near 0 to 1, as shown in Figure S11, while |*r_lr_* | remains unchanged under RCP wave illumination. Notably, the polarization states remain unchanged under varying transmission amplitudes (Figure 2e).

The phase (0–2π) of the transmitted component is modulated by adjusting the propagation length (*u*) of the stripline in the third metallic layer, which, as shown in Figure 2f, provides a full 0–2π phase shift without affecting polarization states (Figure S13b,c). We note that, although amplitude control and phase control are independent, parameter adjustments of stripline/C‐slots may perturb the phase/amplitude profile. Therefore, it is necessary to establish a meta‐atom database to simultaneously fulfill requirements of amplitude (0–1) and phase (0–2π) coverage by co‐optimizing the radius (*r*_1_ and *r*_7_) and gap width (*w*_1_ and *w*_2_) of C‐slot resonators and the length (*u*) of stripline (see Section S5 for detailed comparison). This co‐optimization strategy yields an amplitude–phase library covering approximately 80% of the complex plane, with coverage concentrated in the high‐amplitude regime (>0.7), which fully supports the beam‐steering and polarization‐routing functionalities demonstrated in the following. As a conceptual extension, we propose a dual‑C‑slot design that offers an additional degree of freedom for phase‑independent amplitude tuning, see Figure S14a, which would provide a potential pathway toward full complex‑plane coverage in future implementations.

By integrating polarization routing with independent amplitude, phase, and polarization control within each meta‐atom, our meta‐atom overcomes the limitations of conventional spin‐locked or symmetry‐restricted designs. As can be verified from Figure S15, the meta‑atom exhibits a well‑controlled linear dispersion over a bandwidth of 0.34 GHz (centered at 10.4 GHz). Within this regime, the ellipticity angle variations, polarization‑angle deviation, amplitude fluctuations, and phase errors are confined to within ± 6.3°, below 10°, under 0.1°, and within ±40°, respectively, while the phase preserves 270° coverage. This bandwidth fully accommodates that of the narrowband beam‑steering and polarization‑routing functionalities. To validate this full‐parameter control, we designed two functional metadevices: a polarization‐routing transmissive‐reflective holography and a polarization‐routing asymmetric beamformer, both demonstrating complete three‐dimensional wavefront manipulation.

### 2.3 Experimental Demonstrations

#### 2.3.1 Polarization‐Routing Transmissive‐Reflective Holography

We first experimentally demonstrated a metadevice that supports orthogonal CP decomposition and routing via transmissive‐reflective holography. When the polarization state of the receiving patch is configured in CP state, its independent rotation *ζ*_1_ does not alter the original polarization state while introducing PB phases in cross‐polarized components in reflection and in all transmission components. The corresponding Jones matrix components *J_r_* and *J_t_* are as follows, Equation (2), which support amplitude‐phase modulation in transmission and phase modulation in reflection channels. RCP component of the incident wave is efficiently routed to the reflection channel and phase‐modulated to form a reflective hologram, while the orthogonal polarized (LCP) component is transmitted with simultaneous complex amplitude control, generating a distinct holographic image in the transmission channel. Thus, when the receiving patches are CP states, a polarization‐routing transmissive–reflective holography can be realized.

**FIGURE 3:**
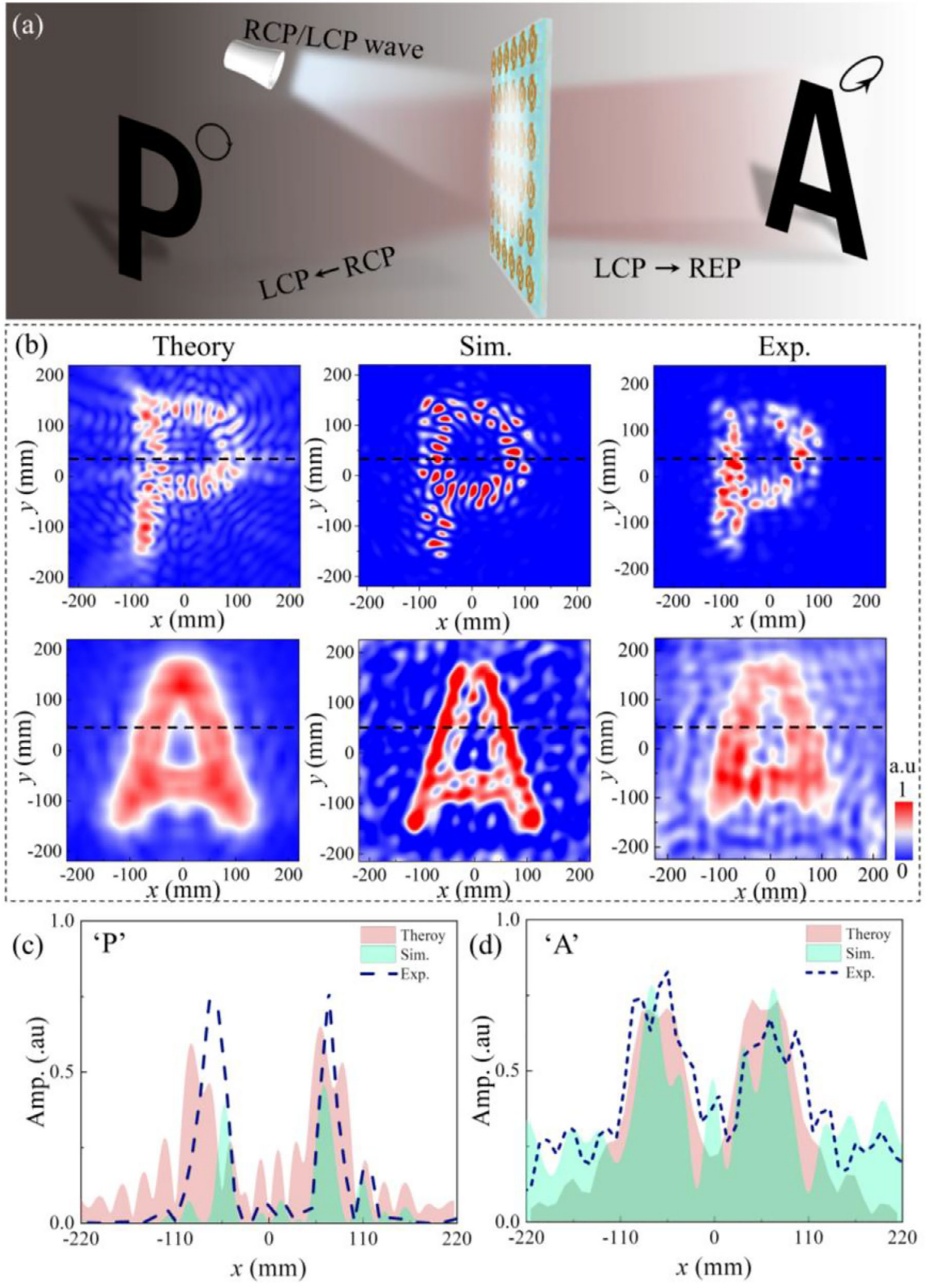
Experimental characterization of polarization‐routing transmissive‐reflective holography. (a) Conceptual demonstration of the metadevice showing LCP‐to‐REP ‘A’ hologram generation in transmission and RCP‐to‐LCP ‘P’ hologram generation in reflection. (b) Electric‐field intensity distribution in transmission and reflection space. (c) Normalized electric‐field intensity profiles of holograms ‘P’ and (d) ‘A’ along the dashed lines marked in (b).

As a representative example, we design a transmissive‐reflective holographic metadevice composed of 44 × 44 meta‐atoms (Figure S11a–d). Each meta‐atom integrates an LCP receiver and a right‐handed elliptically polarized (REP) transmitter with *χ* = 20° and *ψ* = 0°. Accordingly, the angular parameters are set as *γ*_1_ = 22.5°, *ζ*_1_ = 0° and *γ*_2_ = −45°. Rotation of the entire receiving patch (*ζ*_2_) yields an RCP‐to‐LCP holographic profile in reflection space, while tuning the C‐slot dimensions (*r*_1_, *r*_7_, and *ratio*) in the receiving and transmitting patches enables the formation of a complex‐amplitude hologram with arbitrary polarization in transmission. Under RCP illumination, tThe metadevice produces an LCP phase‐only hologram in reflection (Figure 3a). Under LCP illumination, the metadevice generates a REP complex‐amplitude hologram in transmission space.

This polarization‐routing transmissive‐reflective holography is optimized based on the Rayleigh‐Sommerfeld method (see Section S6). The global rotation angles of the receiving patch are optimized to introduce PB phase shifts to the reflected LCP components for generating a hologram (e.g., the letter ‘P’) under RCP incidence. Meanwhile, the geometric parameters of the transmitting and receiving patches and stripline are optimized to coordinately modulate the amplitude, phase, and polarization, generating a holographic image (e.g., the letter ‘A’) in transmission under LCP wave illumination. Experimental results obtained from a fabricated prototype validate the proposed design. As shown in Figure 3b, under 10.3 GHz LCP illumination, an elliptically polarized (*χ* = 20°, *ψ* = 0°) holographic image of the letter ‘A’ is reconstructed in the *xoy‐*plane at *z_t_* = 100 mm. Under 10 GHz RCP incidence, the letter ‘P’ appears in the *xoy‐*plane at *z_r_* = −150 mm. The normalized electric‐field intensity profiles along the dashed lines in Figure 3b exhibit excellent agreement between experimental measurement and theoretical/simulated results (Figure 3c,d), confirming the accuracy and multifunctionality of the designed metadevice.

#### 2.3.2 Polarization‐Routing Asymmetric Beamformer

**FIGURE 4:**
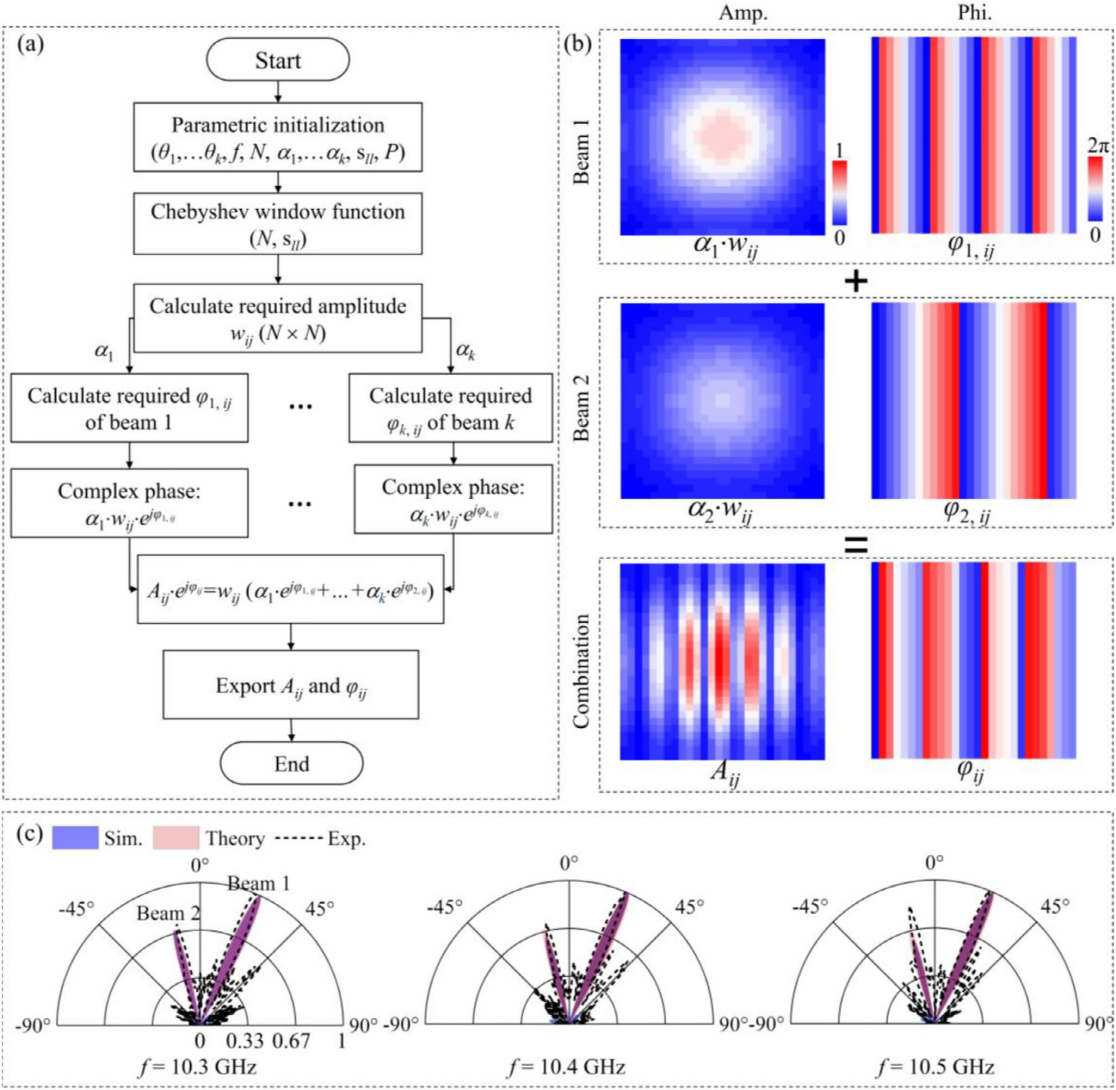
Experimental characterization of the polarization‐routing asymmetric beamformer with different power ratios and deflection angles. (a) Flowchart illustrating the synthesis of the asymmetric beamformer, characterized by the array size (*N *× *N* = 28 × 28), deflection angles (*θ_k_*), frequency (*f*), sidelobe level (S*_ll_*), and beam weighting coefficients (*α_k_*). (b) Amplitude and phase distributions for the two beams on the metasurface plane, wherein the sign “+” represents complex‐phase addition. (c) Calculated, simulated, and measured normalized far‐field radiation patterns at *f* = 10.3, 10.4, and 10.5 GHz, respectively.

The second metadevice that functions as a polarization‐routing asymmetric beamformer is designed for wireless communication between a base station and multiple users located at distinct positions and possessing arbitrary polarization states. Composed of a 28 × 28 array, each meta‐atom integrates an LCP receiver and an RCP transmitter. The beamformer reflects the RCP component of the incident wave while converting the LCP component emitted from the base station into RCP waves directed toward the users, thereby generating two asymmetric beams in transmission with customized power ratios and deflection angles. To realize multi‐beam radiation, we proposed the synthesis approach shown in Figure 4a to calculate the complex‐amplitude coefficient $A_{ij}=\sum_{k=1}^{n}w_{ij}\alpha_{k}e^{j\varphi_{k,ij}}$ (*i* = 1, 2, …, 28, *j* = 1, 2, …, 28). As an illustrative case, we designed asymmetric two beams with relative amplitude weights *α*_1_ = 0.4 and *α*_2_ = 0.6, and deflection angles *θ*_1_ = −15° and *θ*_2_ = 25°. The required phase profile (Figure 4b) for the *ij*‐th meta‐atom was then derived from generalized Snell's law φ_*k*,*ij*_ = −*k*_0_ *x_ij_* sin θ_*k*_, where *x_ij_* represents the position of the *i*‐*j*‐th meta‐atom, *θ_k_* denotes the target deflection angle, and *w_ij_* is the Chebyshev amplitude weighting factor (Figure 4b) used to suppress sidelobes. Experimental far‐field electric intensity measurements of the fabricated prototype validate the performance of this metadevice (see Figure S16). Two RCP scattered beams are observed at deflection angles of 23°/−13°, 23°/−13°, and 22°/−12° with corresponding power ratios of 0.42:0.58, 0.419:0.58, and 0.45:0.55 at *f* = 10.3, 10.4, and 10.5 GHz, respectively (Figure 4c). The measured results exhibit excellent agreement with theoretical calculations and full‐wave simulations, validating the high‐efficiency of the metadevice. The observed beam‐angle deviation of about 2° (measured −13°/23° and designed −15°/25°) is attributed to frequency dispersion, mutual coupling, and inherent fabrication tolerances. Despite this, these angular errors remain within an acceptable level and can be further compensated through closed‑loop calibration algorithms that iteratively adjust the phase gradient based on real‑time beam‑direction feedback.

### 2.4 Potential Applications in Communications

**FIGURE 5:**
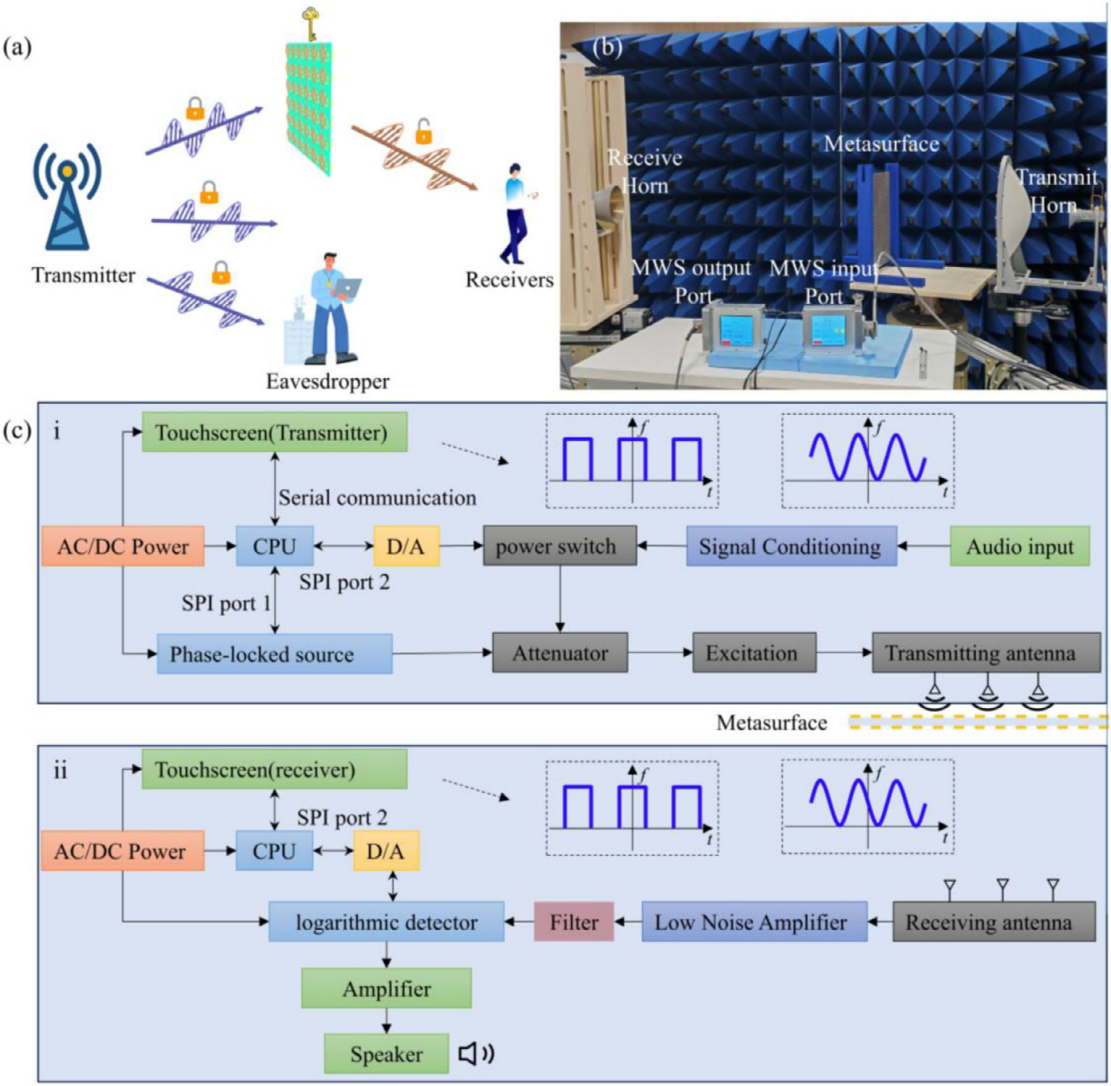
Illustration of potential applications of the polarization‐routing asymmetric beamformer in a communication system. (a) Schematic illustration. (b) Experimental setup. (c) Detailed hardware block diagrams of the (i) MWS transmitter and (ii) receiver.

We now present an experimental setup to demonstrate the potential of our polarization‐routing asymmetric beamformer in communication applications (Figure 5a). As shown in Figure 5b, the experimental setup consists of a digital microwave spectrometer (MWS), a pair of LCP and RCP horn antennas, and the metadevice. MWS converts real‐time text or audio data into modulated microwave signals through a standard AM‐based architecture. Detailed hardware implementation and signal‐processing workflow are provided in Section S7. The radio frequency signal radiated by the LCP horn antenna is directed onto the metadevice, which simultaneously reshapes the amplitude, phase, and polarization of the EM wave. RCP horn receivers positioned along the designed beam directions of −15° and 25° establish real‐time communication links. Owing to the polarization selectivity and wavefront shaping of the metadevice, the system effectively suppresses unintended reception under orthogonal circular polarization states, thus strongly enhancing communication security. Measured received‐signal levels (RSLs) validate the directional dependence of the link performance. Off‐axis receivers detect only weak signals (≈ 1037 mV), which are insufficient for stable data transmission, whereas receivers aligned with the target beams register RSLs above 2300 mV and maintain continuous text and audio communication. The experimental demonstration Video S1 further confirms the successful real‐time transmission of digital patterns (e.g., “*2025*”), text messages, and sine‐wave or square‐wave signals. These results verify that the polarization‐routing asymmetric beamformer enables direction‐selective and polarization‐multiplexed wireless communication, highlighting its potential for advanced communication systems.

## 3 Conclusion

To summarize, this work demonstrates a receiver‐transmitter‐integrated metasurface for polarization decomposing and routing capable of independent and continuous control of amplitude, phase, and polarization over the entire full Poincaré sphere. By tailoring the in‐plane symmetries of the transmitting and receiving patches, the proposed design breaks the conventional orthogonality constraint between input and output polarization states. The metasurface achieves arbitrary polarization conversion with an efficiency exceeding 80.4% between arbitrary two polarization states. To the best of our knowledge, this study demonstrates the first realization of polarization routing and full three‐dimensional EM manipulation with a subwavelength metasurface, fundamentally addressing long‐standing challenges of inter‐dimensional coupling in multifunctional design. The method was experimentally validated through a polarization‐routing transmissive‐reflective holography and an asymmetric beamformer (0.42:0.58 power ratio), confirming the feasibility of high‐efficiency multidimensional control. A proof‐of‐concept wireless communication link incorporating an asymmetric beamformer further confirms its practical potential. Overall, this work establishes a unified platform for multidimensional manipulation of EM waves, paving the way for transformative advancements in future vectorial beam shaping and next‐generation joint sensing‐communications systems.

## 4 Experimental Methods

The metasurfaces were computationally designed and characterized through CST Microwave Studio simulations employing Floquet ports with open boundary conditions. Polarization‐routing transmissive‐reflective holographic metadevice (440 × 440 mm^2^) and asymmetric beamformer (280 × 280 mm^2^) prototypes were fabricated via standard printed circuit board (PCB) processing (fabrication flow process illustrated in Figure S18). The far‐field and near‐field intensity distribution was measured by an AV3672B Vector Network Analyzer (VNA). For near‐field holographic characterization, LCP/RCP plane‐wave feed horns were positioned at distances of 1550 mm/1600 mm, respectively, to ensure plane‐wave illumination. A linearly polarized rectangular waveguide probe scanned a 450 × 450 mm^2^ area in the *xoy*‐plane to capture the EM field distributions at the transmission port (*z_t_* = 100 mm) and the reflection port (*z_r_* = −150 mm) (Figure S17e,f). The elliptical polarization states were reconstructed from two orthogonal components (E*_x_* and E*_y_*) via rotating the waveguide probe by 0° and 90° in two sequential scans. For the far‐field measurement, an LCP plane‐wave feed horn was fixed at *z* = −700 mm, while an RCP receiving horn was positioned at *z* = 1800 mm. The receiver was swept over an angular range of −90°–90° to record the far‐field radiation patterns (see Figure S17a).

## Funding

This work is supported by the National Natural Science Foundation of China (62571546), Xi'an Young and Middle‐aged Leading Scientific and Technological Talents (2025JH‐ZQRC‐0039), Innovative Talents Cultivate Program for Technology Innovation Team of Shaanxi Province (2024RS‐CXTD‐08), Innovation Capability Support Program of Shaanxi (2025ZC‐KJXX‐81), Research Program Project of Youth Innovation Team of Shaanxi Provincial Education Department (24JP221), Youth Innovation Team of Shaanxi Universities, and the Fundamental Research funds of ShaanXi Key Lab of Artificially‐Structured Functional Materials (AFMD‐KFJJ‐23208).

## Conflicts of Interest

The authors declare no conflicts of interest.

## Supporting information




**Supporting File 1**: advs74853‐sup‐0001‐Movie.mp4.

**Supporting File 2**: advs74853‐sup‐0002‐SuppMat.docx.

## Data Availability

The data that support the findings of this study are available in the supplementary material of this article.
