# Supplementary material for: Arbitrary Orthogonal Polarization Decomposition and Routing With Complex Amplitude Modulation via Wheel‐of‐Fortune‐Inspired Metasurfaces
Source: Adv Sci (Weinh). 2026 Mar 17;13(30):e00046. doi: 10.1002/advs.202600046 (PMC13248823; doi:10.1002/advs.202600046)
Supplement: Supplementary file 2 — Supporting File 2: advs74853‐sup‐0002‐SuppMat.docx. [file ADVS-13-e00046-s001.docx]

# Supporting Information

Arbitrary Orthogonal Polarization Decomposition and Routing with Complex Amplitude Modulation via Wheel-of-Fortune-inspired Metasurfaces

*Tong Liu, Changhong Dai, Weike Feng, Yanzhao Wang, Wenxin Liu, Wentao Zhang, Chao Tian, Guangwei Hu^*^, He-Xiu Xu^*^*

*^†^ These authors contributed equally to this work.*

*Tong Liu, Weike Feng, Yanzhao Wang, Wenxin Liu, Wentao Zhang, Chao Tian, He-Xiu Xu*

*Air and Missile Defense College, Air Force Engineering University, Xi’an 710051, China*

E-mail: hxxuellen@gmail.com

*Changhong Dai, Guangwei Hu*

*School of Electrical and Electronic Engineering, Nanyang Technological University, Singapore 639798, Singapore*

E-mail: guangwei.hu@ntu.edu.sg.

Keywords: (Arbitrary polarization, Amplitude-phase, Router, Transmitting and receiving patches)

1. Evolution toward full three-dimensional control of the transmitting wave
2. Polarization routing theory based on the Jones matrix
3. Structure and parameter of the proposed receiver-transmitter-integrated meta-atom
4. Polarization manipulation of receiver-transmitter-integrated meta-atom
5. Amplitude and phase manipulation of receiver-transmitter-integrated meta-atom
6. Polarization‑routing transmissive-reflective holography
7. Polarization‑routing asymmetric beamformer and communication via MWS
8. Fabrication process for receiver-transmitter metasurface

# S1. Evolution toward full three-dimensional control of the transmitting wave

The evolution of metasurface control capabilities has advanced from the regulation of one single degree of freedom of transmitting wave—such as phase^[1]^, amplitude^[2]^, or polarization—to the simultaneous manipulation of two independent degrees (e.g., phase and polarization^[3]^, amplitude and polarization^[4]^, or amplitude and phase^[5, 6]^). A key advance in this trajectory is the achievement of fully integrated three-dimensional control over phase, amplitude, and polarization, which enables unprecedented versatility in wavefront engineering. In polarization control, the field has advanced from basic conversions between specific polarization states (such as linear and circular polarization) to independent control over orthogonal polarization states, and further to the generalized manipulation of dual non-orthogonal polarization states^[7]^, as depicted in Fig. S1. The schematic clearly delineates the developmental trajectory from constrained, low-dimensional control to versatile, high-dimensional wave manipulation. The top tier marked “this work” demonstrates a breakthrough in non-orthogonal polarization control, achieving full-parameter, multi-degree-of-freedom governance.

The distinctive advantages of our architecture become evident when compared with available representative platforms in this field (Table S1). A primary advantage lies in its functionally decoupled architecture. The stacked twisted metasurface platform enables arbitrary polarization conversion based on non‑orthogonal eigenstate theory^[7]^. In contrast, our receiver‑transmitter‑integrated metasurface introduces independent control layers. This decoupling enables the independent optimization of the radiation beam without perturbing the polarization‑routing function. Although the polarization conversion efficiency (exceeding 80.4% and peak 94.4%) is somewhat reduced due to insertion loss of vias and bilayer structure, the radiating layer can be freely engineered for beam shaping without affecting the core routing performance. Another key advantage is the inherent polarization insensitivity of the receiving layer. Unlike the multi‑atom metasurface platform^[8]^, which requires numerical optimization for non‑CP wave incidences, our approach is fundamentally independent of the incident polarization state (linear, circular, or elliptical). This fundamental independence arises because the receiving layer performs routing function via its structural symmetry, granting the platform universal applicability to arbitrary polarization states. Rather than optimizing a single performance metric, our work establishes functional decoupling as a new design paradigm. It provides a distinct and versatile architecture for integrated polarization routing and multi‑dimensional wavefront control, thereby complementing and extending prior advances in metasurface design.


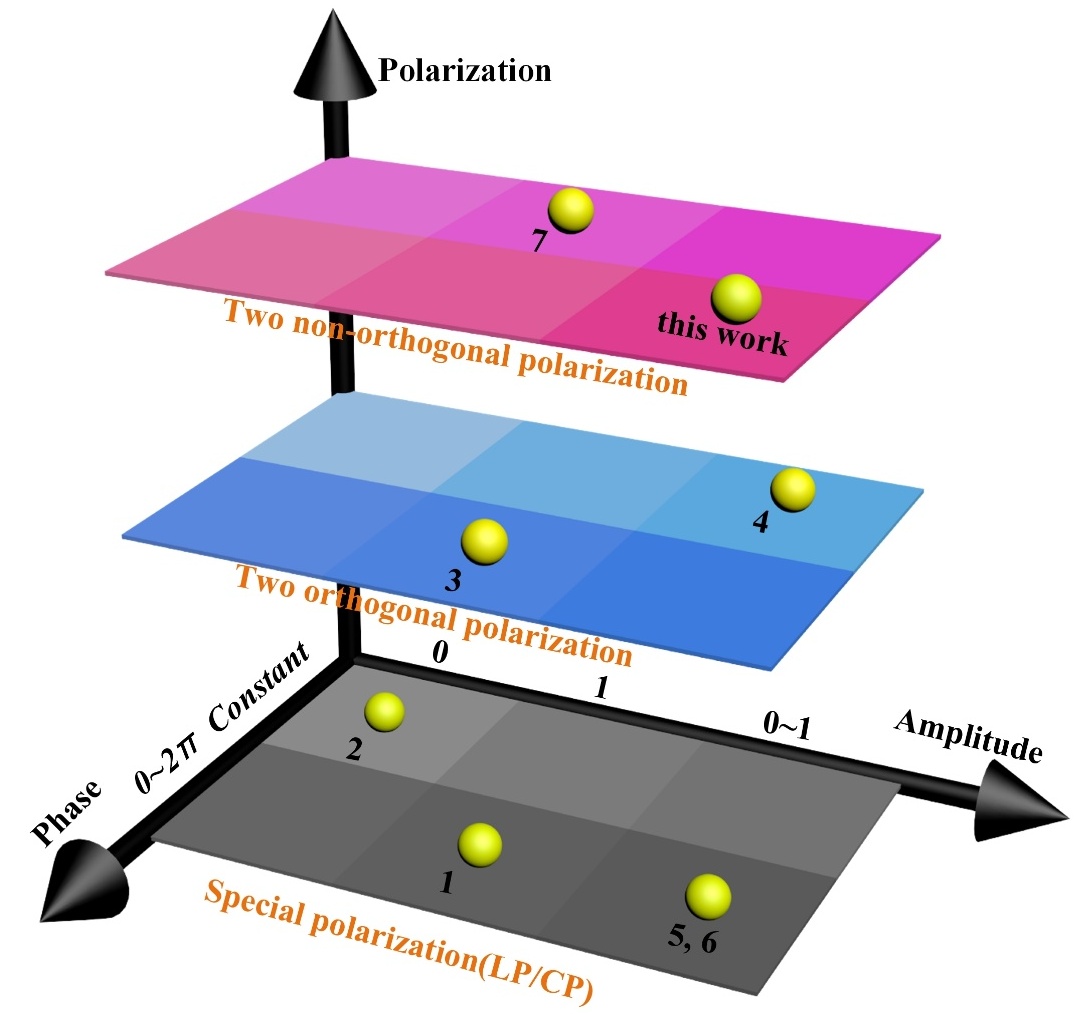


1. Evolution toward full three-dimensional control of amplitude, phase, and polarization of transmitting wave, with representative performance comparisons ^[1-7]^.

Table S1 Comparison of key performance with available state-of-the-art platforms for multi-dimensional wave control.

|  | [7] | [8] | This Work |
| --- | --- | --- | --- |
| Metasurface platform | Stacked twisted metasurface | Multi-atom metasurface | Receiver-transmitter-integrated metasurface |
| Polarization conversion efficiency | Near 100% | Near 100% | ＞80.4% (reach up to 94%) |
| Functionality | Arbitrary polarization conversion | Amplitude, phase, and polarization control | Polarization routing + amplitude, phase, and polarization control |
| Polarization of incident waves | Arbitrary | Analytic solutions are available only for CP | Arbitrary |

**S2. Polarization routing theory based on the Jones matrix**

1. **Polarization decomposition and projection operators**

We begin by establishing a general theoretical framework for polarization routers that enables independent control of the amplitude, phase, and polarization of the output electromagnetic wave. To achieve decomposition and independent routing of arbitrary polarization states, we introduce an orthonormal polarization basis {$|e_{1}\rangle$, $|e_{2}\rangle$} satisfying $|e_{1}\rangle^{\dagger}|e_{2}\rangle=0$. An incident plane wave with a complex electric field ***E****_in_* can be decomposed as:

$E_{in}=a|e_{1}\rangle+b|e_{2}\rangle$. (S1)

The core routing operation‒directing the $|e_{1}\rangle$ component to the transmission port and the $|e_{2}\rangle$ component to the reflection port‒is mathematically described by projection operators:

. (S2)

These operators exhibit idempotent ($P_{i}^{2}=P_{i}$) and Hermitian properties ($P_{1}+P_{2}=I$). To enable independent wavefront modulation of each routed component, we factor the transmission and reflection Jones matrices (***J****_t_* and ***J****_r_*) into a product of a modulation matrix and a projector:

. (S3)

Here, ***U****_t_* and ***U****_r_* are general 2×2 complex matrices that apply linear transformations to the transmitted and reflected fields. For lossless or low-loss operation, ***U*** is typically constituted as a unitary matrix multiplied by a complex amplitude, describing pure polarization transformation alongside overall amplitude and phase shift:

, . (S4)

The coefficients *A_t_*, *A_r_* ∈ [0, 1] control the transition and reflection amplitude, while *ϕ_t_*, *ϕ_r_* ∈ [0, 2π] are corresponding phase delays. The matrices ***R****_t_*, ***R****_r_* ∈ SU(2) are unitary matrices implementing arbitrary polarization transformations, parameterized by the Poincaré sphere coordinates (azimuth angle *ψ*, ellipticity angle *χ*). In the linear polarization basis, these matrices are expressed as:

$R\left(\psi_{t},\chi_{t}\right)=\left(\begin{matrix} R_{11}^{\left(t\right)} & R_{12}^{\left(t\right)} \\ R_{21}^{\left(t\right)} & R_{22}^{\left(t\right)} \end{matrix}\right)$, (S5)

$R\left(\psi_{r},\chi_{r}\right)=\left(\begin{matrix} R_{11}^{\left(r\right)} & R_{12}^{\left(r\right)} \\ R_{21}^{\left(r\right)} & R_{22}^{\left(r\right)} \end{matrix}\right)$. (S6)

Here, $R\left(\psi,\chi\right)$ represents an SU(2) matrix describing the pure polarization transformation on the Poincaré sphere.

For the linear basis vectors $|e_{x}\rangle=\left(\begin{matrix} 1 \\ 0 \end{matrix}\right)$ and $|e_{y}\rangle=\left(\begin{matrix} 0 \\ 1 \end{matrix}\right)$, the projector operators could be simplified to:

$P_{1}=\left(\begin{matrix} 1 & 0 \\ 0 & 0 \end{matrix}\right),$ $P_{2}=\left(\begin{matrix} 0 & 0 \\ 0 & 1 \end{matrix}\right)$. (S7)

For the CP basis vector, we define:$|e_{L}\rangle=\frac{1}{\sqrt{2}}\left(\begin{matrix} 1 \\ i \end{matrix}\right)$ and $|e_{R}\rangle=\frac{1}{\sqrt{2}}\left(\begin{matrix} 1 \\ -i \end{matrix}\right)$. The corresponding projector operators are:

$P_{1}=\frac{1}{2}\left(\begin{matrix} 1 & -i \\ i & 1 \end{matrix}\right)$, $P_{2}=\frac{1}{2}\left(\begin{matrix} 1 & i \\ -i & 1 \end{matrix}\right)$. (S8)

The transformation matrix from the linear basis to the circular basis is:$T=\frac{1}{\sqrt{2}}\left(\begin{matrix} 1 & 1 \\ i & -i \end{matrix}\right),$transforming the projection operator $P_{1}$ and $P_{2}$ from the linear basis to the circular basis:

$P_{1,\text{cir}}=T^{-1}P_{1}T=\left(\begin{matrix} 1 & 0 \\ 0 & 0 \end{matrix}\right)$, $P_{2,\text{cir}}=T^{-1}P_{2}T=\left(\begin{matrix} 0 & 0 \\ 0 & 1 \end{matrix}\right)$. (S9)

The general model then yields the transmission and reflection matrices:

, (S10)

. (S11)

1. **Transmission matrix**

For an incident wave with a routed transmission polarization state, its transmitted field can be expressed as:

, (S12)

where $R_{t}$ is the sole variable determining the polarization state of the transmitted wave. To achieve arbitrary to arbitrary polarization conversion, the SU(2) matrix $R_{t}$ in a receiving and radiating metasurfaces can be decomposed into the product of a geometric‑phase rotation matrix and an ellipticity‑modulation matrix. In the circular polarization basis, the geometric-phase rotation — corresponding to the rotation of the receiving and radiating patch — is described by a diagonal matrix: $R\left(\zeta\right)=\left(\begin{matrix} e^{i\zeta} & 0 \\ 0 & e^{-i\zeta} \end{matrix}\right)$, where *ζ* is the rotation angle of the patch. Here, ellipticity modulation corresponds to a rotation about the S_2_ axis of the Poincaré sphere. In the linear polarization basis, the matrix for a rotation by an angle 2*χ_t_* about the S_2_ axis is $E^{lin}\left(\chi_{t}\right)=\left(\begin{matrix} \text{cos}\chi_{t} & i\text{sin}\chi_{t} \\ i\text{sin}\chi_{t} & \text{cos}\chi_{t} \end{matrix}\right)$, where $\chi_{t}$ is the ellipticity angle. Transforming this matrix into the circular basis via the basis transformation matrix ***T*** yields:

$E^{cir}\left(\chi_{t}\right)=TE^{lin}\left(\chi_{t}\right)T^{-1}=\left(\begin{matrix} \text{cos}\chi_{t} & \text{sin}\chi_{t} \\ -\text{sin}\chi_{t} & \text{cos}\chi_{t} \end{matrix}\right)$. (S13)

This matrix describes a rotation by 2*χ_t_* about the S_2_ axis in the circular basis, controlling the amplitude ratio between the right-handed circularly polarized (RCP) and left-handed circularly polarized (LCP) components. Ellipticity modulation can be implemented using anisotropic resonant patches, where the aspect ratio of the patch governs $\chi_{t}$.

The most general polarization transformation achievable with a bilayer (receiving and radiating) metasurfaces is the product of a receiving-layer rotation, a radiating-layer ellipticity modulation, and a radiating-layer rotation:

$R_{r}^{cir}=R\left(\zeta_{1}\right)E^{cir}\left(\chi_{t}\right)R\left(\zeta_{2}\right)$ (S14)

Here, $\zeta_{1}$ and $\zeta_{2}$ are the geometric phase angles introduced by the radiating and receiving layers, respectively. The three real parameters $\left(\zeta_{1},\chi_{t},\zeta_{2}\right)$ span the entire SU(2) group, enabling arbitrary polarization conversion between any two points on the Poincaré sphere. Including the overall amplitude and propagation phase, the complete Jones matrix for the transmission components can be written as

. (S15)

Substituting this expression into the general transmission matrix yields:

, (S16)

where $A_{t}\in\left[0,1\right]$ is the transmission coefficient and is the propagation phase. For the routing transmission polarization wave $E_{in}=(1,0)^{\text{T}}$, the transmitted field is modified to

. (S17)

This expression shows that the two geometric‑phase angles $\zeta_{1}$ and $\zeta_{2}$ act independently. The sum $\zeta_{1}+\zeta_{2}$ determines the phase of the co-polarized component, and the difference $\zeta_{1}-\zeta_{2}$ determines the phase of the cross-polarized component. Together with the ellipticity angle $\chi_{t}$ (controlling the amplitude ratio between the two components), the transmission coefficient $A_{t}$, and the propagation phase , the model provides a complete set of independent parameters for full three-dimensional control of the transmitted wave. This parameterization offers a clear, physically intuitive guideline for the design of multilayer receiving-radiating metasurfaces with arbitrary polarization routing functionality.

1. **Reflection matrix**
2. **Reflection matrix in the linear polarization basis**

We consider a metasurface router designed to transmit the *x*-LP component and reflect *the y-*LP component. Under the symmetry constraint *r_xy_* = *r_yx_*, the reflection matrix remains diagonal. In a more general scenario, the transmission amplitude can be continuously tuned from 0 to 1, such as patch-type meta-atoms via impedance matching. When *t_xx_* = 0, the matrix $\boldsymbol{R}_{r}$ reduces to a general SU(2) matrix, with the unitary component taking the diagonal form $R_{r}=\left(\begin{matrix} e^{i\beta} & 0 \\ 0 & e^{-i\beta} \end{matrix}\right)$. The general form of the reflection matrix is therefore, where $A_{r}=1$ and denotes a relative phase difference between the two orthogonal reflection components.

For *t_xx_* ≠ 0, energy conservation imposes $\left|t_{xx}|^{2}+\right|t_{yx}|^{2}+|r_{xx}|^{2}=1$. Mapping the transmission matrix onto the model form , we obtain the relations and . In the LP basis, the reflected wave maintains the same polarization as the incident wave. The energy conservation equation for the *x*-LP wave input then becomes:

$\text{∣}t_{xx}\text{∣}^{2}+\text{∣}t_{yx}\text{∣}^{2}+\text{∣}r_{xx}\text{∣}^{2}=A_{t}^{2}(\text{∣}R_{11}^{\left(t\right)}\text{∣}^{2}|+\text{∣}R_{21}^{\left(t\right)}\text{∣}^{2})+A_{r1}^{2}\text{∣}R_{11}^{\left(r1\right)}\text{∣}^{2}=1$, (S18)

which leads to a complementary relation between the amplitude coefficients: $A_{t}^{2}+A_{r}^{2}=1$. This result indicates that the transmission coefficient *A_t_* and the co-polarized reflection coefficient *A_r_* vary in a complementary manner. For *y*-LP incidence, the reflection characteristics determined by the patch size remain unchanged, i.e., $r_{yy}=1$, $r_{xy}=0$. Consequently, the reflection matrix can be expressed as:

. (S19)

Assuming and , the corresponding reflection and transmission matrices take the form . This functional dependency holds universally for arbitrary orthogonal polarized states‒including linear, circular, and elliptical states‒ because the underlying energy-conservation condition, symmetry constraints, and the projection-operator formalism are all independent of the chosen basis.

1. **Reflection matrix in the circular polarization basis**

The routing target in the circular basis involves primarily transmitting the LCP component while reflecting the RCP component:

. (S20)

Imposing the common symmetrical constraint *r_ll_* *= r_rr_*, the target reflection matrix takes the form:$J_{r}^{cir}=\left(\begin{matrix} 0 & r_{lr} \\ 0 & 0 \end{matrix}\right)$, where *r_lr_* is the complex reflection coefficient with |*r_lr_*| = 1. Substituting into the general model and solving for the modulation matrix (an SU(2) matrix satisfying $R^{\dagger}R=I$ and $\text{det}\left(R\right)=1$) yields:

$R_{r}^{cir}=\left(\begin{matrix} 0 & e^{i\beta} \\ -e^{-i\beta} & 0 \end{matrix}\right)$, (S21)

where $-e^{-i\beta}$ can be written as $e^{i\pi}\cdot e^{-i\beta}=e^{i\left(\pi-\beta\right)}$, corresponding to a phase delay of π. Since the polarization state of the receiving layer is defined in the circularly polarized (CP) basis and rotated by an angle *ζ*_2_, this rotation introduces a geometric phase that modifies the phase of the reflection coefficients without altering the polarization selectivity. Its Jones matrix can be expressed as:

(S22)

Substituting back into the model gives:

. (S23)

This model describes an ideal polarization router that completely routes RCP waves to reflection while directing LCP waves to transmission. When the transmission amplitude is tuned via the impedance of the radiating patch, the geometry and rotation angle of the receiving patch remain unchanged. Consequently, the reflection coefficient for LCP waves is no longer zero, whereas the form of the reflection matrix for RCP waves remains unaltered. The reflection matrix takes the general form: with the composite phase terms given by and , where and denote the initial phase differences induced by the geometric structural parameters. For a pure structural rotation (i.e., without modifying other parameters), the corresponding Jones matrix reduces to: $U_{r}\text{ }=\left[\begin{matrix} 0 & e^{i(t_{2}-2\zeta_{2})} \\ A_{r}e^{i(t_{1}+2\zeta_{2})} & 0 \end{matrix}\right]$.

# S3. Structure and parameter of the proposed receiver-transmitter-integratedmeta-atom

To ensure that manipulation of the radiating patch does not interfere with the polarization‑routing function of the receiving patch, a metallized background is introduced within the stacked structure. The resonance and scattering fields of the receiving patch are primarily concentrated above the upper ground plane, while the near‑field energy of the radiating patch is strictly confined below the lower ground plane or localized around specific coupling slots. This physical isolation ensures that surface current distribution on the receiving patch is dictated solely by the incident wave polarization and remains independent of the state of the lower radiating patch. Polarization selectivity is achieved by independently controlling the structural symmetry of the receiving patch, whereas the symmetry of the radiating patch exclusively controls the transmitted wave polarization.

The proposed meta-atom for polarization, phase, and amplitude manipulation features a periodicity of *p_x_* = *p_y_* =10 mm, comprising five metallic layers separated by four dielectric substrates (total thickness of 2.5mm), as shown in Fig. S2. The first and fourth dielectric layers utilize F4BM350 (*h*_1_ = 2 mm, *ε*_r_ = 3.5, tan*δ* = 0.001), and the second and third dielectric substrates employ thinner F4BM265 (*h*_2_ = 0.2 mm, *ε*_r_ = 25, tan*δ* = 0.015). The top and bottom metallic layers function as receiving and transmitting patches, respectively, and are interconnected through anti-symmetrically arranged metallized vias and striplines. Two pairs of arc-shaped slots are etched along the periphery of each patch, with an angular spacing of 180° within each pair and a 90° rotational offset between the two pairs. The C-slot resonators in receiving and transmitting patches are dimensioned through proportional scaling (*w*_1_ = *r*_1_/*ratio* for receiving patches and *w*_2_ = *r*_7_/3 for transmitting patches), where parameter ‘*ratio*’ denotes the diameter-to-width proportionality in receiving patches. Phase modulation at transmission components is precisely controlled by adjusting the propagation path length between the receiving and transmitting patches, achieved through a stripline and antisymmetric metallic via configuration oriented from (*l_x_*, *l_y_*) to (-*l_x_*, -*l_y_*), enabling tunable phase shifts according to Δ*ϕ* = *βl*, where *β* is the propagation constant, *l_x_* = *l_y_* = 0.5mm.


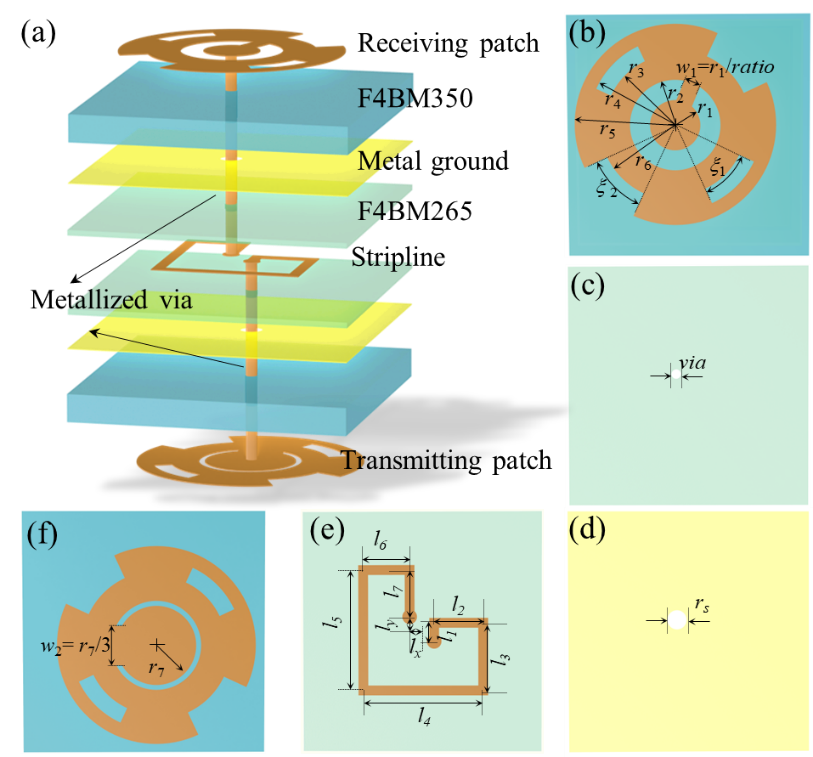


1. Wheel-of-Fortune-inspired meta-atom and its structural components. (a) The structure of the Wheel-of-Fortune-inspired meta-atom comprises receiving and transmitting patches, F4BM350 and F4BM265 substrates, stripline, metallic background, and via. (b) Structure of receiving patch integrates a C-slot resonator and bisymmetric split-ring elements for polarization state manipulation, where *r*_1_ = mm, *r*_2_ = 3 mm, *r*_3_ = 2.8 mm, *r*_4_ = 3.45 mm, *r*_5_ = 4 mm, *r*_6_ = 3 mm, and *α*_1_ = *α*_2_ = 40°. (c) Dielectric substrate with circular-slotted vias (*via* = 0.2 mm). (d) Metallic background with circular slots (*r_s_* = 0.3mm). (e) Stripline facilitating phase manipulation across the operational bandwidth (characterized by width *w*_3_ and total length *l*, $l=\sum_{i=1}^{7} l_{i}=8u+13.5via$, where *l*_1_ = *u* + 2*via*, *l*_2_ = 4 *u* + *via*, *l*_3_ = 5*u* + 2*via*, *l*_4_ = 8*u* + 4.5*via*, *l*_5_ = 8 *u* + 4.5*via*, *l*_6_ = 4 *u* + 0.5 *via*, *l*_7_ = 4*u* - 0.5*via*. (f) Structure of transmitting patches, where the inner radius and opening width of the C-slot resonator are *r*_7_ and *w_2_ =r*_7_/3.

To quantify the isolation, we perform full‑wave simulations. Figs S3 and S4 show the surface current distributions on the receiving patch under LCP and RCP incidence, respectively, for varying *ζ*_1_ from -45° to 45° in steps of 22.5°, with *γ*_1_=0°. The results confirm that the current distributions remain nearly unchanged regardless of variations in the lower‑layer control parameters, indicating that the polarization identification function is preserved. Furthermore, we further evaluate the scattering parameters under RCP and LCP incidence for different stripline length coefficients *u* and ellipticity angles *γ*_2_. Fig. S5 plots the transmission and reflection coefficients as functions of *ζ*_1_ (varied from -45° to 45° in steps of 10°, γ_1_=0°), while Fig. S5b shows the same coefficients as functions of *u* (varied from 0.2 to 0.9 in steps of 0.1, with *ζ*_1_ = -45° and *γ*_1_=0°). At the resonant frequency (*f* = 10 GHz), the total transmission ($|E_{T}|=\sqrt{\left|T_{ll}\right|^{2}+\left|T_{rl}\right|^{2}}$) and reflection coefficients ($|E_{R}|=\sqrt{\left|R_{lr}\right|^{2}+\left|R_{rr}\right|^{2}}$) exhibit only minimal fluctuations, confirming that manipulation of the transmitted component does not significantly perturb the polarization‑routing function.


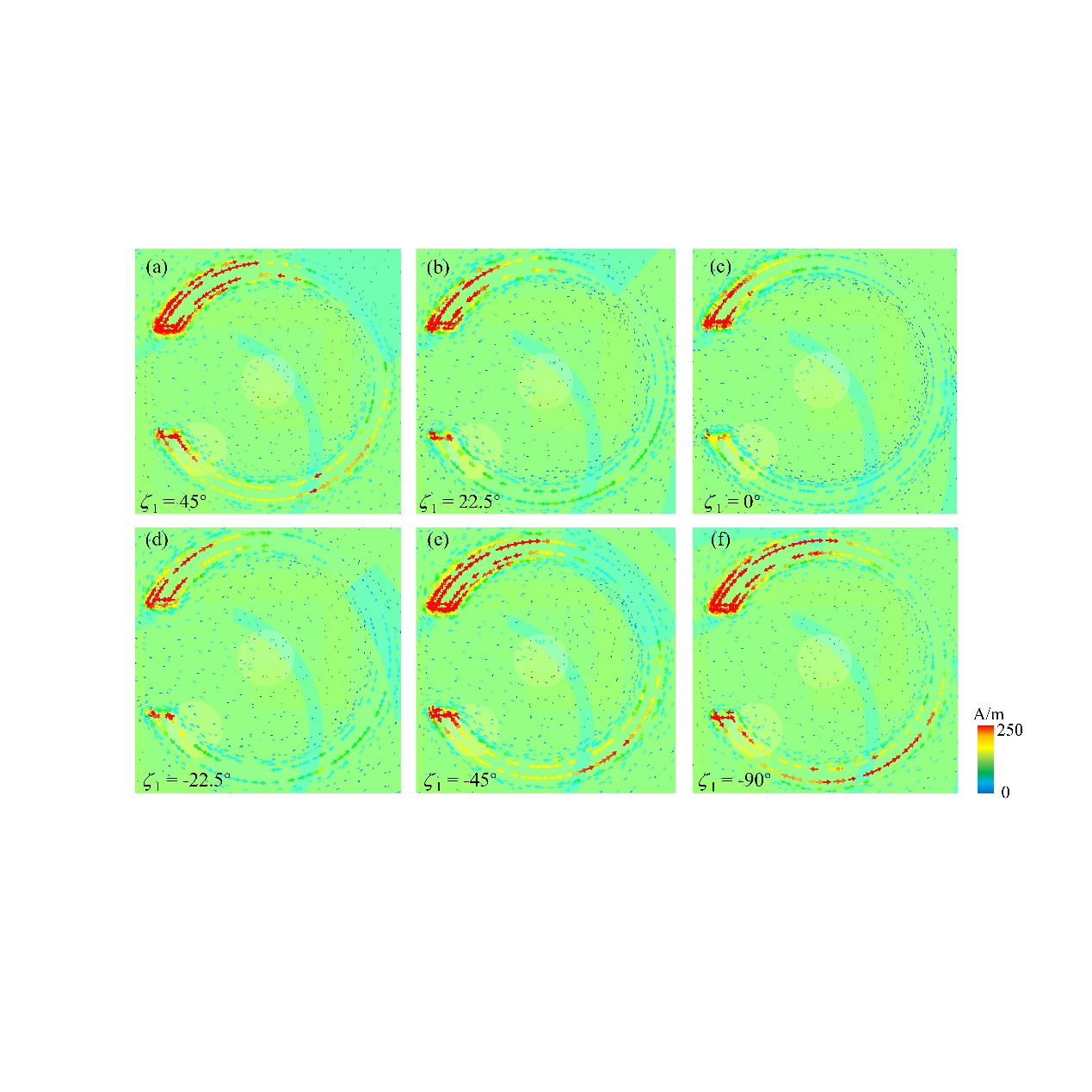


1. (a-f) Surface current distributions on receiving patches across varying *ζ*_1_ from -45° to 45° in steps of 22.5° under LCP wave incidence, with *γ*_1_ fixed at 0°.


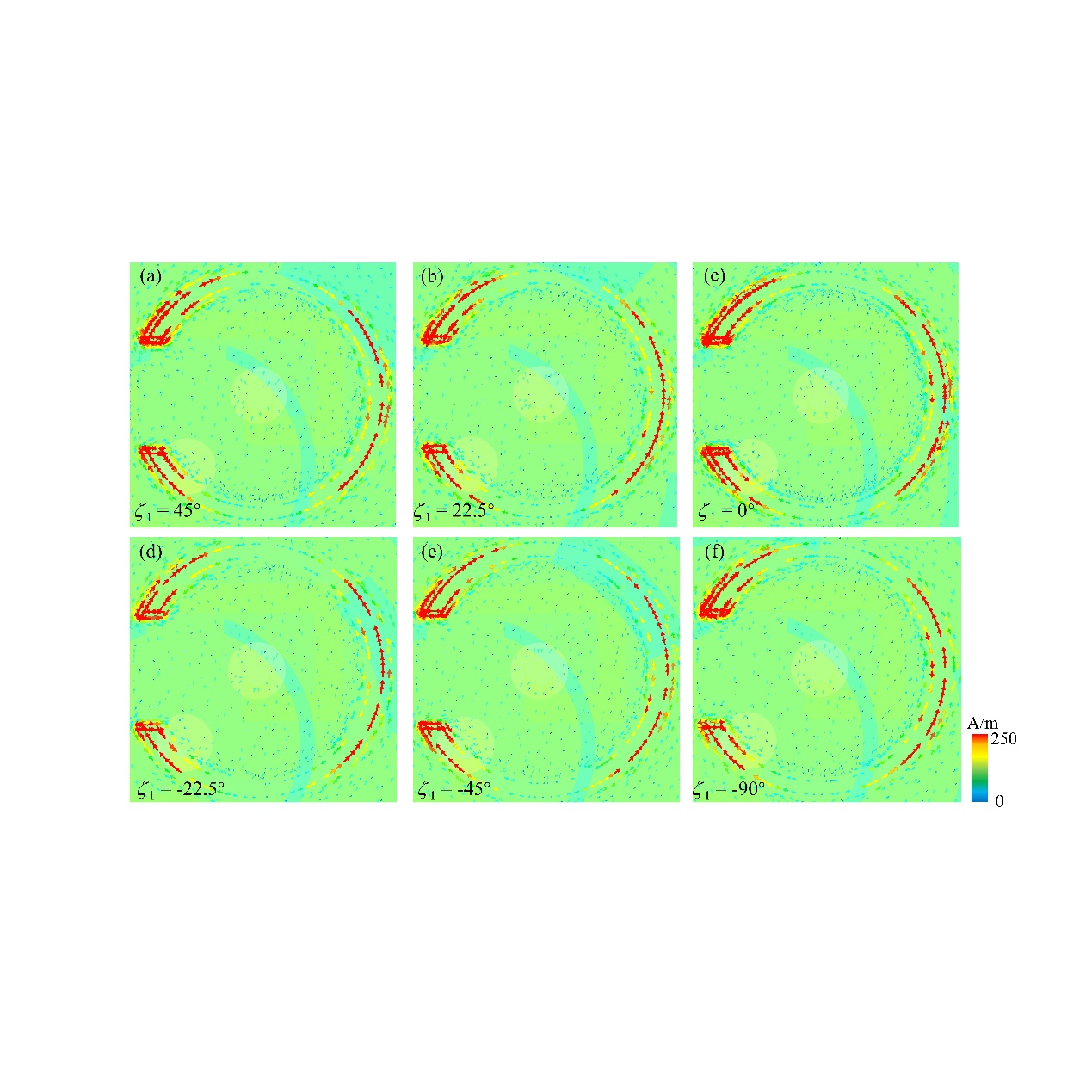


1. (a-f) Surface current distributions on receiving patches across varying *ζ*_1_ from -45° to 45° in steps of 22.5° under RCP wave incidence, with *γ*_1_ fixed at 0°.





1. (a) Transmission and reflection coefficients as a function of *ζ*_1_, varied from -45° to 45° in steps of 10°, with *γ*_1_ fixed at 0°. (b) Transmission and reflection coefficients as a function of the parameter *u*, varied from 0.2 to 0.9 in steps of 0.1, with*ζ*_1_ = -45° and *γ*_1_ = 0°.

# S4. Polarization manipulation of receiver-transmitter-integrated meta-atom

The manipulation of polarization encompasses precise control over both ellipticity angle (*χ*) and polarization orientation angle (*ψ*) of EM waves, which could be achieved by adjusting the relative rotational angle *γ* between C-slots and bisymmetric split-ring resonators and globe rotational angle *ζ* of whole transmitting/receiving patches. This adjustment breaks the in-plane symmetry of transmitting patches, thereby flexibility modulates the amplitude ratio of LCP and RCP components to manipulate the ellipticity angle *χ***—**validated through surface current distributions and orthogonal circular polarized components versus angle *γ*_1_ (Figs. S6-S9). The polarization orientation angle is dynamically adjusted by clockwise rotating the receive/radiate patch around the center of the metallized vias, introducing a phase difference between LCP and RCP components. As derived from theoretical analysis, the relationship between polarization angle and rotation angle is *ψ* = *ζ*_1_. Finally, continuous polarization transformation from RCP (*γ* = 45°) to LCP wave (*γ* = -45°) via elliptical states is achieved.

The relationship between polarization state (*χ*, *ψ*) and geometric parameters is shown in Table S2. Fig S8 presents the variation of *χ*, *ψ*, and transmission amplitude as functions of frequency for different *γ*_1_ values. As *γ*_1_ varies from -45° to 0°, the resonant point shifts to a lower frequency, with *γ*_1_ =0° exhibiting frequency-insensitive behavior, whereas from 0° to 45° the resonant point shifts to a higher frequency. Notably, *ψ* remains nearly constant over 10.3-10.5 GHz. These results confirm that despite the frequency dependence of *χ*, the key polarization control metrics fully satisfy the design requirements within the 10.3-10.5 GHz band. As illustrated in Fig. S9 a, numerical simulations of the meta-atom's polarization and transmission response reveal that variations in *γ*_1_ notably influence the polarization angle *ψ*. This deviation can be compensated by actively tuning *ψ*, which itself does not affect the ellipticity angle *χ* or the transmission coefficient. Moreover, Fig S9c plots the conversion-efficiency as a function of the target output polarization in the Poincaré sphere coordinates (2*ψ*, 2*χ*) under LCP incidence. The results show that the efficiency varies significantly with the ellipticity angle 2*χ* (controlled by *γ*_1_), while remaining nearly constant with respect to changes in the polarization angle 2*ψ* (controlled by *ζ*_1_), reflecting the rotational symmetry of the structure. Furthermore, for various output polarizations, including LCP, LECP, *x*-LP, RECP, and RCP, the peak conversion efficiencies all exceed 80.4% and can reach up to 94.4%, demonstrating that the proposed framework achieves high-efficiency polarization conversion under diverse output polarizations.

Table S2 The relationship between the polarization state and its corresponding geometric parameters

| Polarization states(*χ*, *ψ*) | Geometry parameters | |
| --- | --- | --- |
|  | relative rotational angle *γ* | globe rotational angle *ζ* |
| LP | *γ =* 0° | *ψ* (0° ~ 360°) |
| LHEP | -45° < *γ* < 0° | *ψ* (0° ~ 360°) |
| RHEP | 0° < *γ* < 45° | *ψ* (0° ~ 360°) |
| LCP | *γ =* -45° | *ψ* (0° ~ 360°) |
| RCP | *γ =* 45° | *ψ* (0° ~ 360°) |


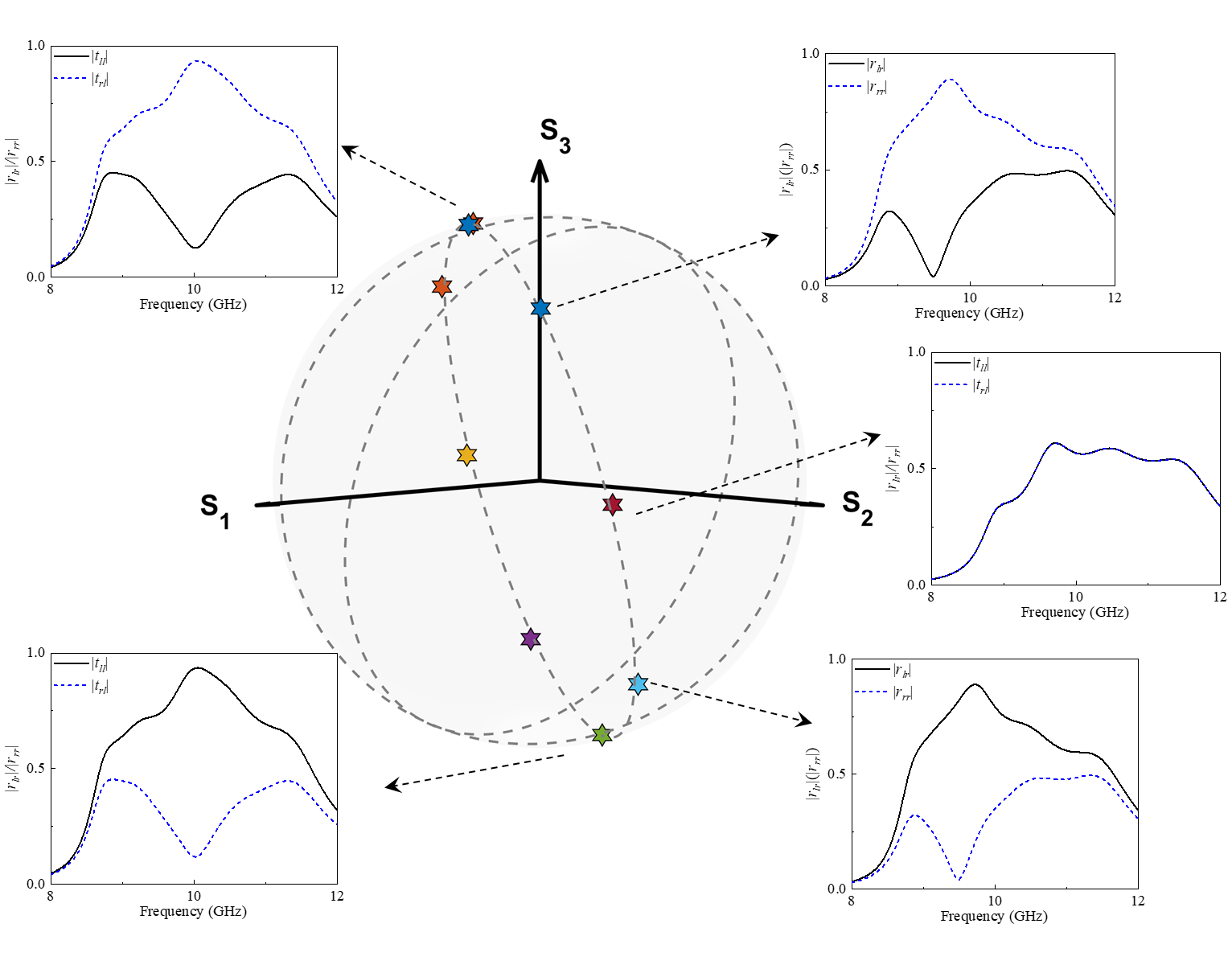


1. Characterization of polarization states on the Poincaré sphere and corresponding amplitude distributions of LCP/RCP components on transmitting patches for different local rotation angles.


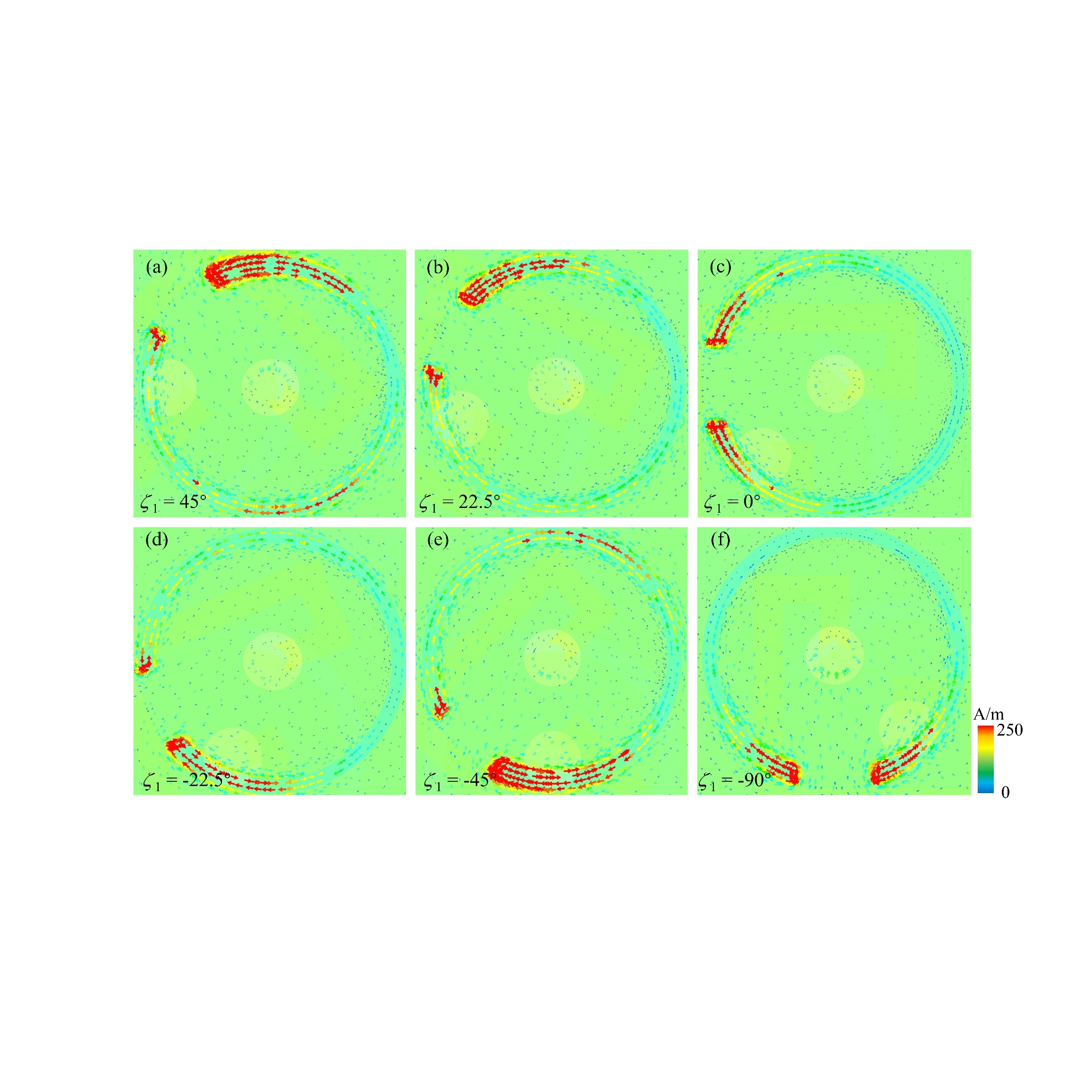


1. (a-f) Surface current distributions on transmitting patches across varying *ζ*_1_ from -45° to 45° in steps of 22.5°, with *γ*_1_ fixed at 0°.


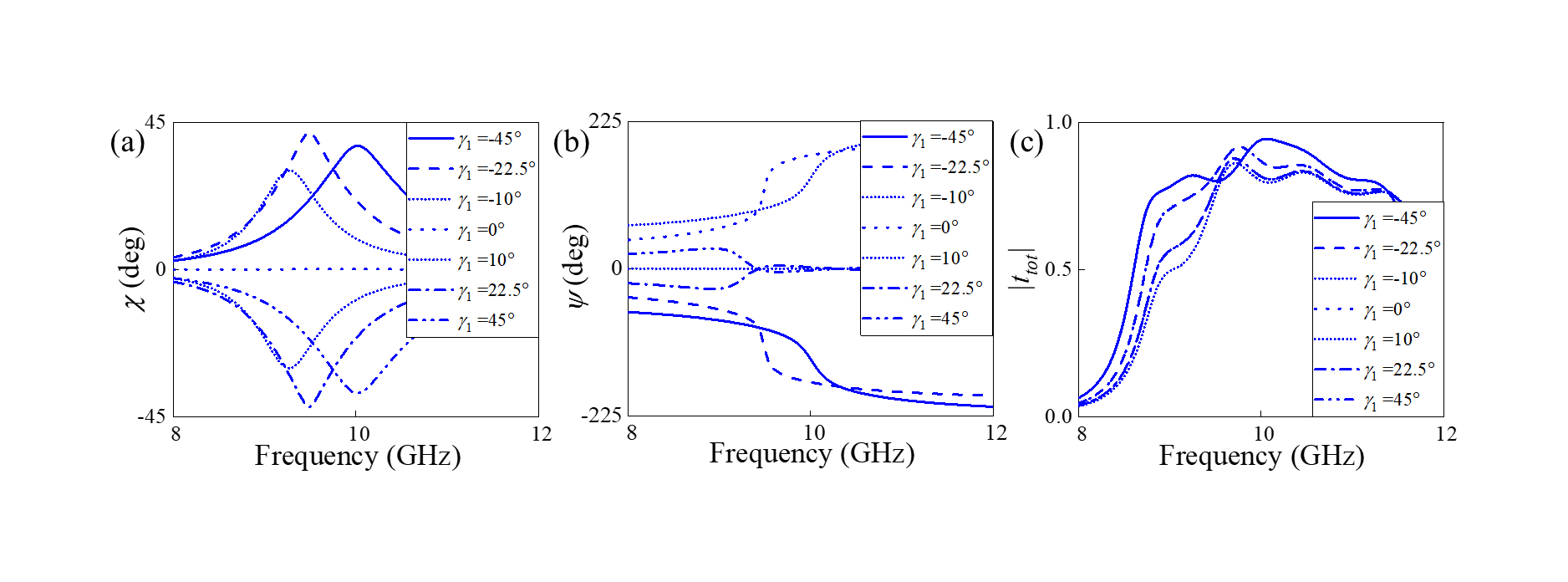


1. Polarization response and transmission response characterization of the meta-atom. (a) Ellipticity angle *χ*, (b) polarization angle *ψ*, and (c) total transmission coefficient as functions of the rotation angle *γ*_1_ across -45° to 45° range.


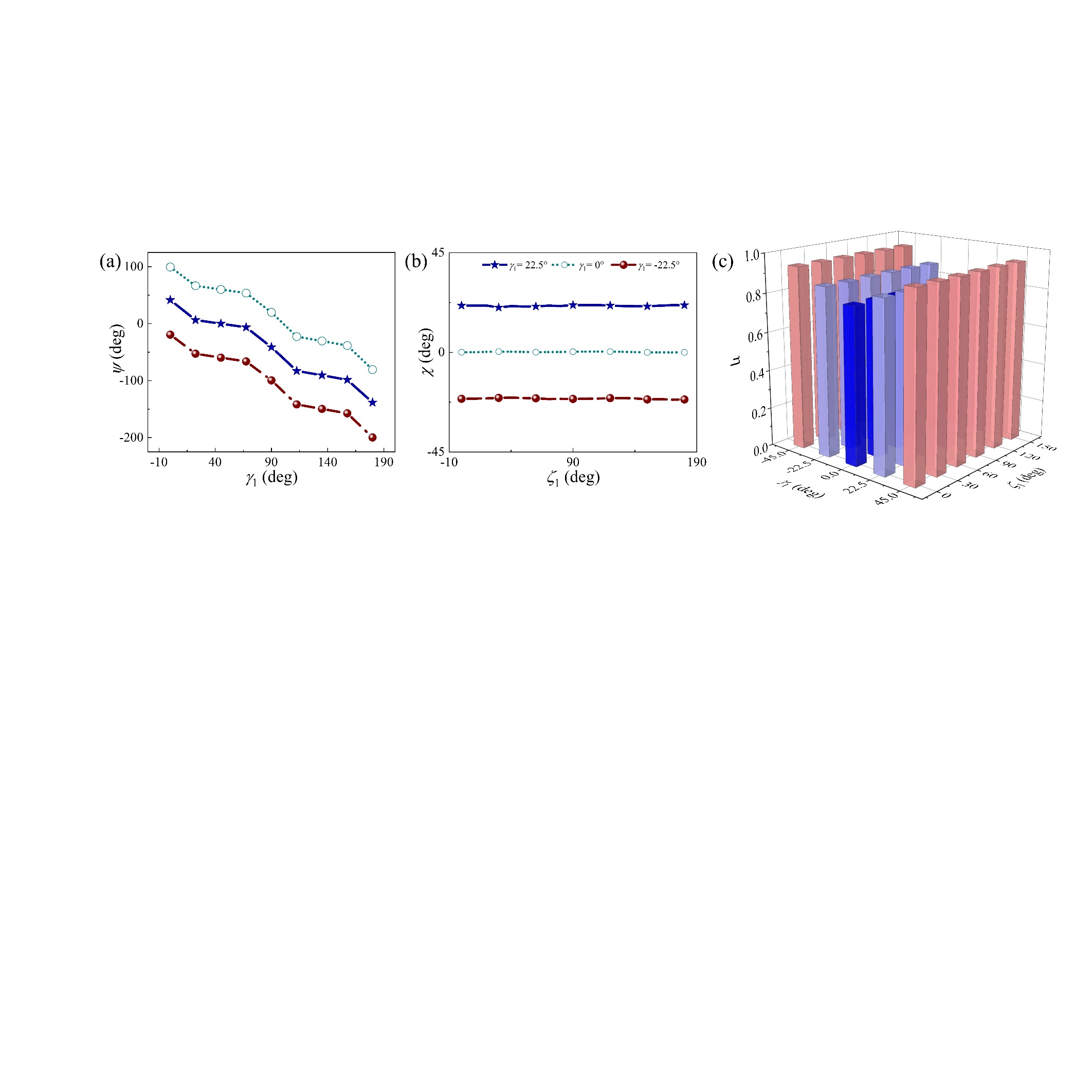


1. Characterization of polarization states and conversion efficiency versus geometric parameters (*γ*_1_ and *ζ*_1_) at 10 GHz. (a) Azimuth angle *ψ* versus local rotation angle *γ*_1_ (22.5° step) for *ζ*_1_ = 0°, 60°, and 120°. (b) *χ* versus global rotation angle *ζ*_1_ (30° step) for *γ*_1_ = 22.5°, 0°, and -22.5°. (c) Conversion efficiency of the designed meta-atom under different geometric parameters *γ*_1_ and *ζ*_1_, which govern the ellipticity angle *χ* and the principal axis angle *ψ*, respectively.


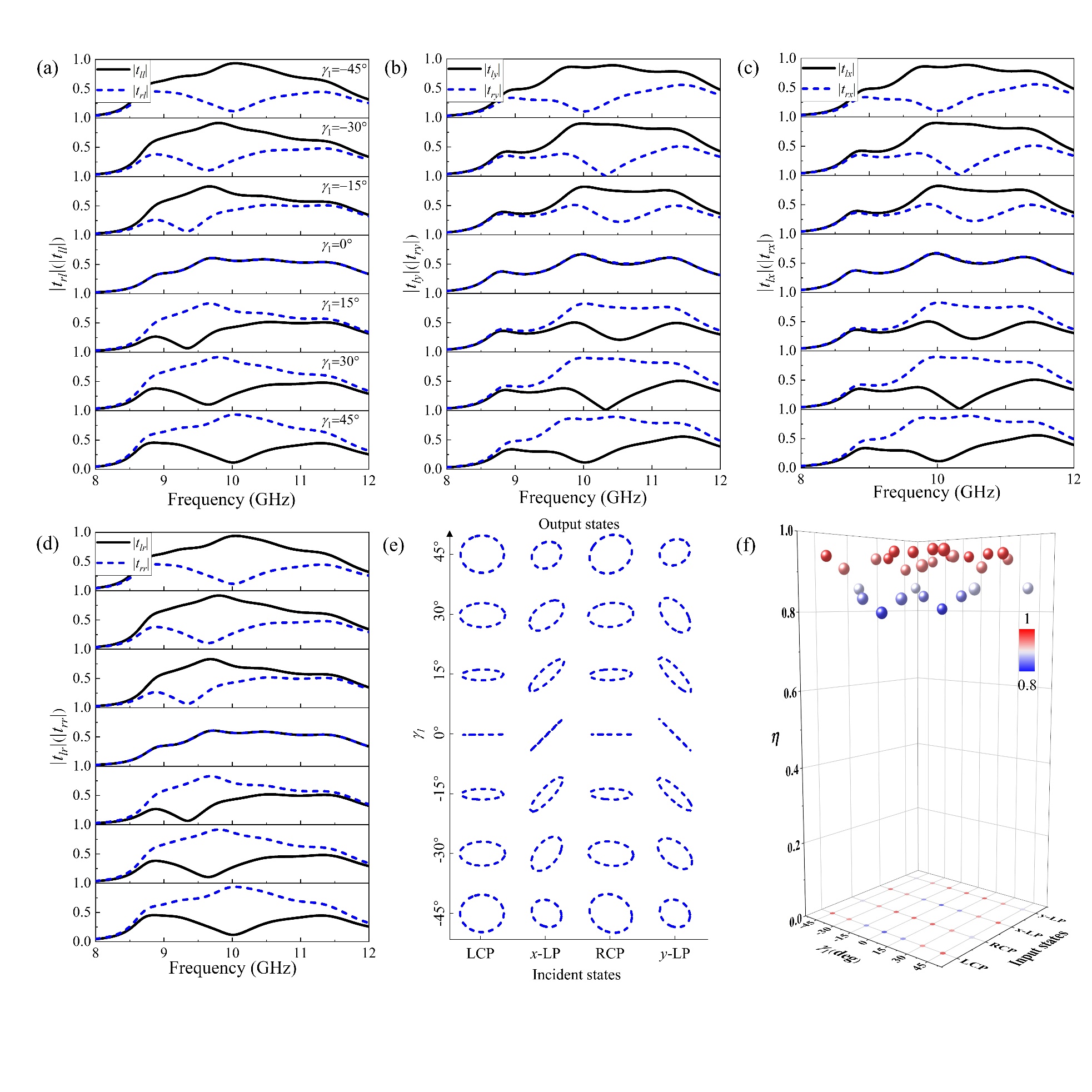


1. Amplitude and polarization profiles of two orthogonal circular polarized components of a meta-atom with variable γ_1_ of radiation patch from -45° to 45° under the illumination of (a) LCP, (b) *x-*LP, (c) *y-*LP, and (d) RCP wave. For each incident wave, the geometric parameters of the receiving patch are configured as follows: (*γ*_2_ = 0°, *ξ*_2_ = 0°) for *x*-LP incidence, (*γ*_2_ = 0°, *ξ*_2_ = 90°) for *y*-LP incidence, and (*γ*_2_ = 45°, *ξ*_2_ = 0°) for RCP incidence. (e) Evolution of the output polarization state and (f) the corresponding conversion efficiency as a function of the radiation patch rotation angle *γ*_1_ from -45° to 45° for the meta-atom.

To systematically evaluate the polarization-manipulation performance under illumination by incident waves with different polarization states—*x*‑LP, *y*‑LP, LCP, and RCP—the receiving patch is configured accordingly (*γ*_2_ = 0°, *ζ*_2_ = 0° for *x*‑LP; *γ*_2_ = 0°, *ζ*_2_ = 90° for *y*‑LP; *γ*_2_ = 45°, *ζ*_2_ = 0° for RCP), while the radiating patch rotation angle *γ*_1_ is varied continuously from -45° to 45° in steps of 15°. As shown in Fig. S10 a-d, the amplitude distributions of the orthogonal circularly polarized components exhibit a dependence on *γ*₁: the LCP component decreases from near unity to nearly zero as *γ*_1_ increases, while the RCP component shows the opposite trend, with equal amplitudes at *γ*_1_ = 0° corresponding to linear polarization. Based on these responses, we calculate the output polarization state at 10 GHz and plot its evolution on the Poincaré sphere (Fig. S10 e). As *γ*_1_ varies from -45° to 45°, the output state follows a continuous great‑circle trajectory, transitioning from LCP through elliptical states to RCP, thereby accessing a continuous range of elliptical states covering the full transition between the two circular polarizations. The total transmission efficiency—defined as $|E_{T}|=\sqrt{\left|T_{ll}\right|^{2}+\left|T_{rl}\right|^{2}}$ —remains consistently above 80.4% across the entire tuning range (Fig. S10f), confirming that high performance is maintained while achieving continuous polarization tunability.

# S5. Amplitude and phase manipulation of receiver-transmitter-integrated meta-atom

Modifying the C-slot resonator dimensions facilitates precise transmission amplitude control. Simultaneously reducing slot width (*w*_1_ and *w*_2_) and inner diameter (*r*_1_ and *r*_7_) from 1.5 mm and 3.2 mm to 0 mm achieves a transmission amplitude decrease from near-unity to complete suppression. Energy redistribution between transmitted components and reflected cross-polarized elements follows the relationship established in Section S1. Therefore, the cross-polarized reflection coefficient remains stable, as evidenced by the amplitude of relationships across reflection spaces in Fig. S11.


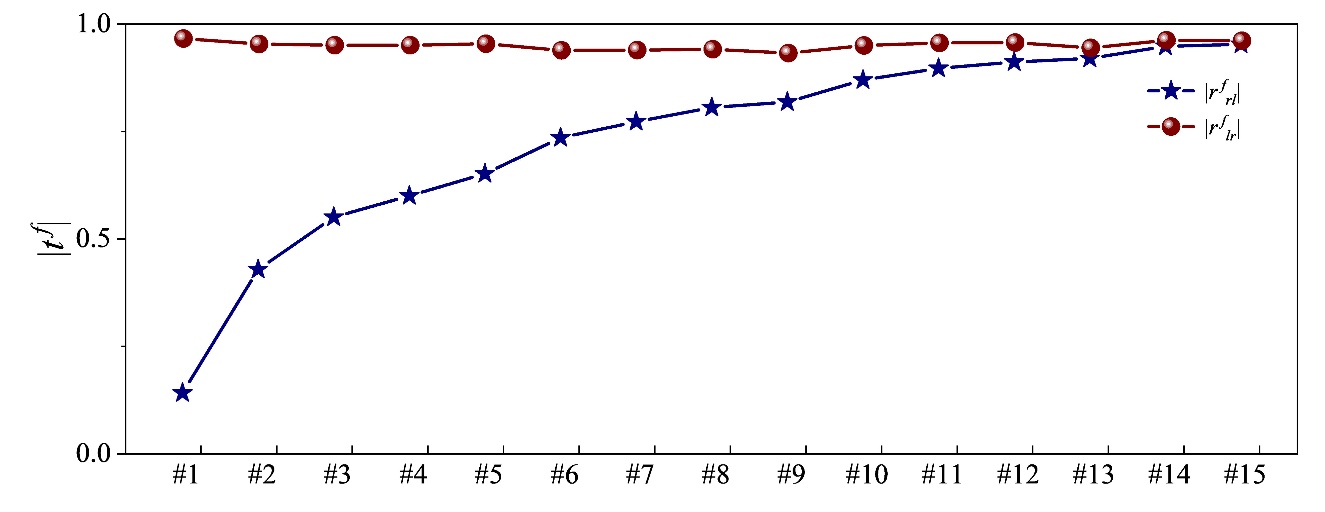


1. Reflection amplitude responses of cross-polarized components versus varying C-slot dimensions.

Table S3 Transmission coefficients versus different parameters of C-slot resonators of receiving and transmitting patches

| Number | *r*_1_ (mm) | *r*_7_ (mm) | *ratio* | *u* |
| --- | --- | --- | --- | --- |
| #1 | 3 | 0.72 | 6.3 | 0.35 |
| #2 | 3 | 3.2 | 2 | 0.35 |
| #3 | 3 | 0.1 | 150 | 0.35 |
| #4 | 3 | 0.2 | 8 | 0.35 |
| #5 | 1.4 | 3.2 | 10 | 0.35 |
| #6 | 2.6 | 3.2 | 10 | 0.35 |
| #7 | 3 | 0.1 | 15 | 0.35 |
| #8 | 3 | 0.72 | 15 | 0.35 |
| #9 | 1 | 3.2 | 10.67 | 0.35 |
| #10 | 1.5 | 3.2 | 6 | 0.35 |
| #11 | 2.6 | 3.2 | 4 | 0.35 |
| #12 | 2.2 | 3.2 | 2 | 0.35 |
| #13 | 2.6 | 3.2 | 2 | 0.35 |
| #14 | 3 | 0.1 | 6 | 0.35 |
| #15 | 3 | 0.1 | 10 | 0.35 |

To validate phase coverage characteristics, we constructed a meta-atom and systematically analyzed its phase and amplitude response under LCP and RCP wave illumination (Fig. S12). Simulated results demonstrate full 2π phase coverage in the transmission components via adjustment of stripline length parameter *u*, while maintaining near-zero phase shifts in reflection components. However, this change in electrical length inevitably alters the input impedance seen from the receiving patch port, which may lead to impedance mismatch and reduce the transmission efficiency of the routed polarization component. To compensate for this effect, we employ a co-optimization strategy: for each target phase (i.e., each specific *u*), we simultaneously optimize the geometrical dimensions of the receiving and radiating patches (*r*_1_, *r*_7_, and *ratio*). This dynamic compensation mitigates impedance variations while achieving the desired phase shift, enabling the construction of a comprehensive amplitude-phase library for each polarization channel, as shown in Fig. S13. The library covers approximately 80% of the target complex plane, with the coverage concentrated in the high‑amplitude region (>0.7), which is sufficient to realize the beam‑steering and polarization‑routing functionalities demonstrated in this work. The 20% unattainable states are predominantly located in the low-amplitude regime, where impedance mismatch arising from C-slot parameter variations and the specific stripline lengths required for extreme phase values prevent these amplitude-phase combinations from being included in the library.


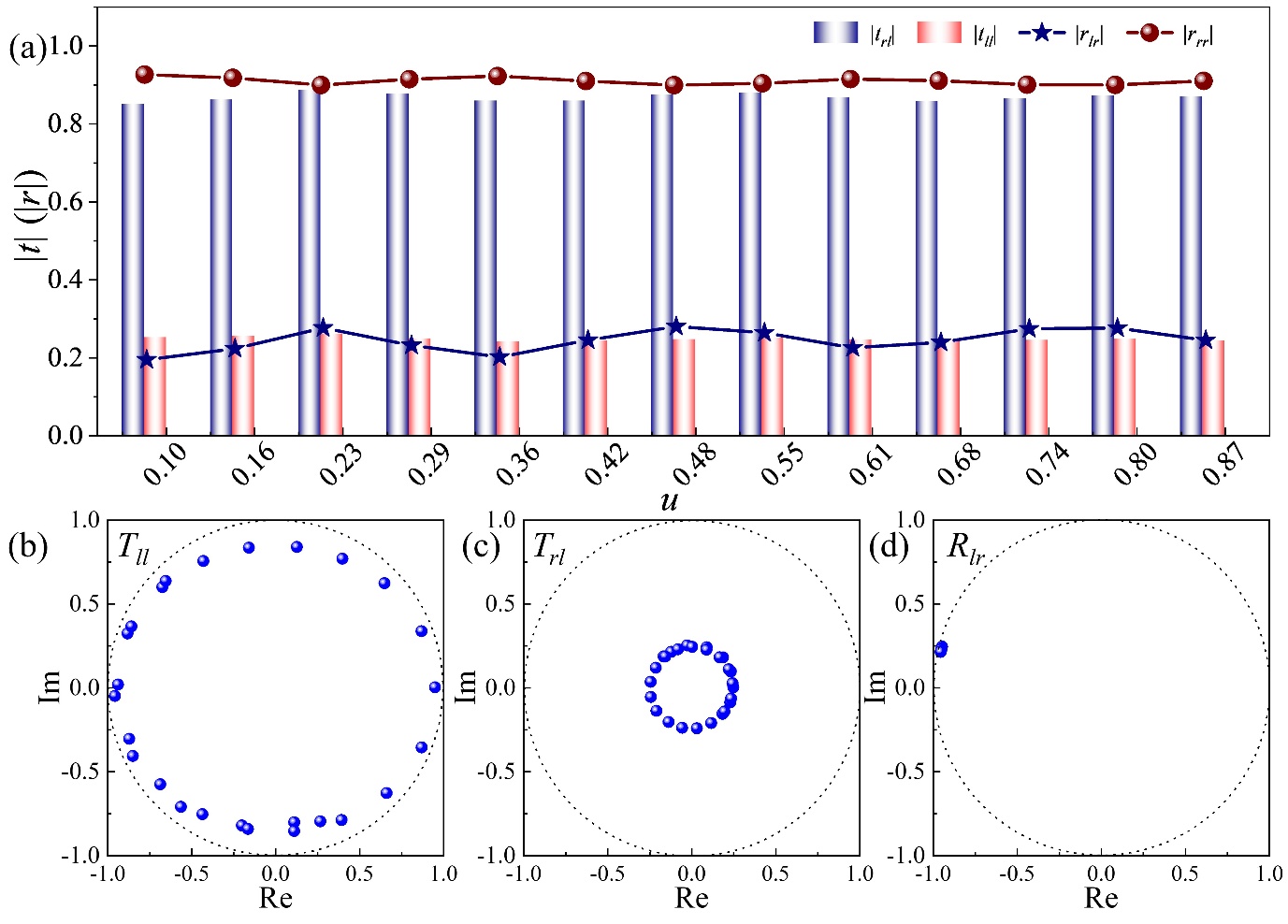


1. Additional results of amplitude response and complex-field distributions for the meta-atom with different *u* values. (a) Transmission and reflection amplitude profiles versus *u* values with fixed parameters *r*_1_ = *r*_7_ = 3.2 mm, *ratio* = 3. Normalized distribution of transmission components (b) *T_ll_* and (c) *T_rl_*, and (d) reflection components *T_lr_* in complex amplitude space.


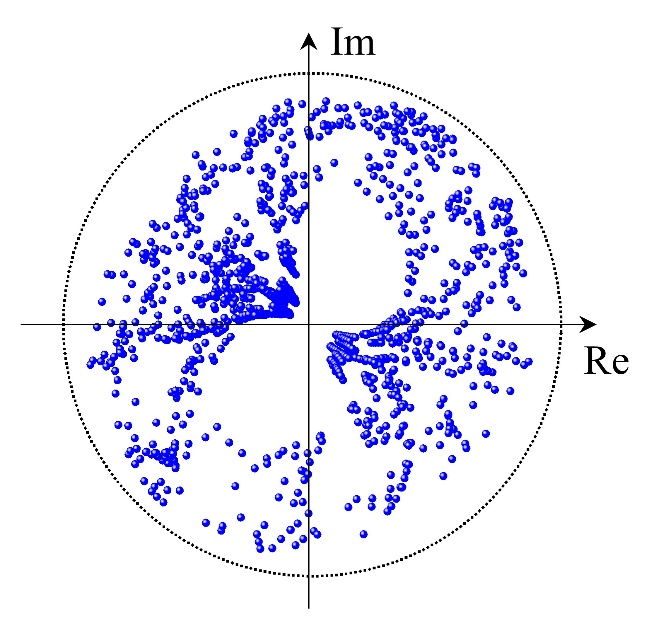


1. The distribution of transmission components of established databases in complex amplitude space.

To extend coverage into the low‑amplitude region while preserving phase controllability, we introduce a dual‑C‑slot design (Fig. S14a) featuring two metallic connecting rods with independently tunable opening angles. The relative rotation angle provides an additional amplitude‑tuning degree of freedom without perturbing the phase response. Preliminary results (Fig. 14b) demonstrate that this approach populates previously inaccessible low‑amplitude states, complementing the dense high‑amplitude coverage shown in the complex‑plane scatter plot. A detailed study of this enhanced design will be presented in future work.


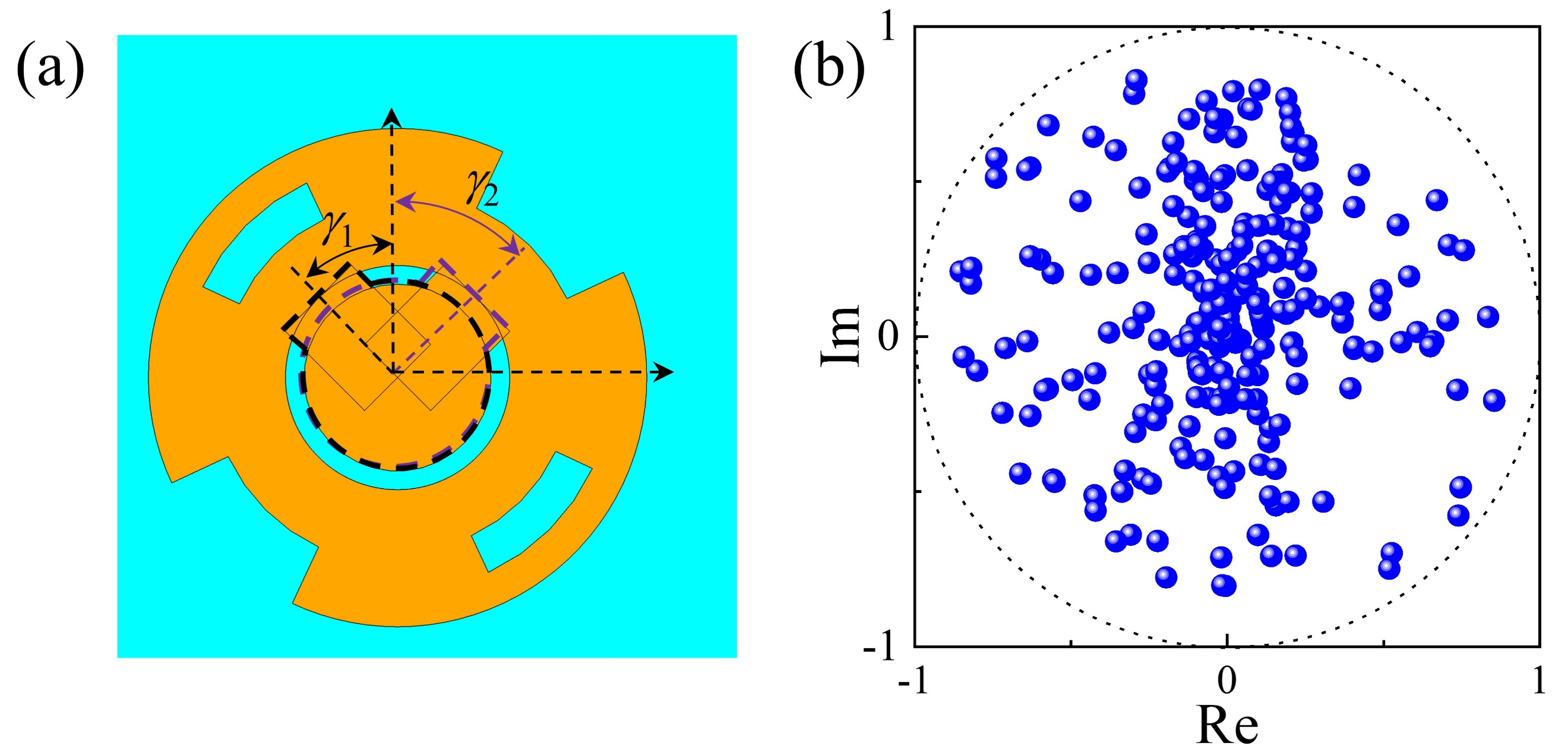


1. Improved unit-cell structure for achieving broader complex-amplitude coverage. (a) Schematic of the proposed dual‑C‑slot configuration, featuring two C-slots with independently adjustable opening angles controlled by orientation angles *ζ*_1_ and *ζ*_2_. (b) Corresponding distribution of the transmission coefficient on the complex plane, showing the real (Re) and imaginary (Im) parts and demonstrating the extended coverage enabled by this proposed design.

To evaluate the broadband potential of the proposed meta-atom, we quantify the frequency-dependent behavior of the key electromagnetic responses-ellipticity angle (*χ*), polarization angle (*ψ*), transmission amplitude, and phase—over the 10.3-10.5 GHz range. The results are summarized in Fig. S15. The ellipticity angle *χ* (controlled by *γ*_1_) exhibits noticeable frequency dependence, reflecting the polarization-sensitive nature of the metasurface unit. At *γ*_1_=0°, the variation in *χ* over the 10.3-10.5 GHz band is only 0.01°, whereas at *γ*_1_ = ±45° the variations reach ±6.3°. In contrast, the polarization-angle error Δ*ψ*_1_ (determined by *ζ*_1_) remains nearly constant across the frequency band, with a deviation of approximately 9.7°. The transmission amplitude, controlled by *r*_1_, *r*_7_, and *ratio*, exhibits fluctuations within 0.034 (linear value), demonstrating good amplitude stability. The transmission phase, tunable via the stripline length coefficient *u*, achieves over 360° phase coverage, with frequency-induced variations ranging from -29.1° to -38.4°.

Based on the above metrics, we define the effective operational bandwidth of the meta‑atom as the frequency range that simultaneously satisfies the following conditions: ellipticity angle error ≤6.3°, polarization angle error ≤10°, amplitude fluctuation ≤0.1 (linear value), and phase error ≤40° while maintaining ≥360° phase coverage. Under this definition, the proposed metaatom exhibits a bandwidth that is sufficient for narrowband applications. We further analyzed the bandwidth under specific amplitude-phase targets. As shown in Figs S15 e and f, with reference targets at 10.4 GHz (amplitudes 0.3-0.9 in steps of 0.1 with tolerance ±0.1, phases 0°-270° in steps of 90° with tolerance ±40°), the resulting amplitude and phase bandwidths demonstrate reliable performance (> 0.34 GHz) across most target combinations.





1. Analysis of the variations of key electromagnetic parameters over the 10.3 - 10.5 GHz range: (a) ellipticity-angle variation Δ*χ*_1_ versus *γ*_1_; (b) polarization-angle variation Δ*ψ*_1_ versus *ζ*_1_; (c) transmission coefficient variation Δ|*t_rl_*| controlled by the amplitude-tuning library; (d) transmission-phase variation Δ*φ_rl_* versus *u*; (e) Bandwidth of the amplitude and phase responses of the meta-atom library under *t_rl_* channel; (f) corresponding amplitude and phase bandwidths centered at 10.4 GHz for these targets defined in (e).

# S6. Polarization‑routing transmissive-reflective holography

The tri-dimensional manipulation performance is experimentally verified through polarization-selective holography in transmission and reflection spaces. In the reflection space, a pure-phase holography was designed based on the PB phase mechanism. Conversely, in the transmission space, a polarization-selective complex-amplitude holography was achieved through our proposed manipulation strategy of amplitude, phase, and polarization. The reconstruction process is governed by the diffraction integral:

$U'\left(x_{0},y_{0}\right)=\frac{1}{j\lambda}\text{∬}U\left(x_{h},y_{h}\right)\frac{e^{ikr}}{r}\text{cos}\cdot\xi dx_{h}dy_{h}$.

where *h* denotes the separation between holographic and imaging planes, with *U*(*x_h_*, *y_h_*) and *U*′(*x*_0_, *y*_0_) representing electric-field distributions at respective planes. The required amplitude and phase profiles of each meta-atom were derived numerically via inverse Fourier transform of the target images (Fig. S16a and b), thereby enabling precise wavefront control. The metasurface model and fabricated prototype are shown in Figs. S17c and d, which were characterized by a near-field experimental setup (Figs. S17e and f). This dual-channel metasurface ensures fully decoupled control over transmitted and reflected wave properties, enabling independent holographic projections.


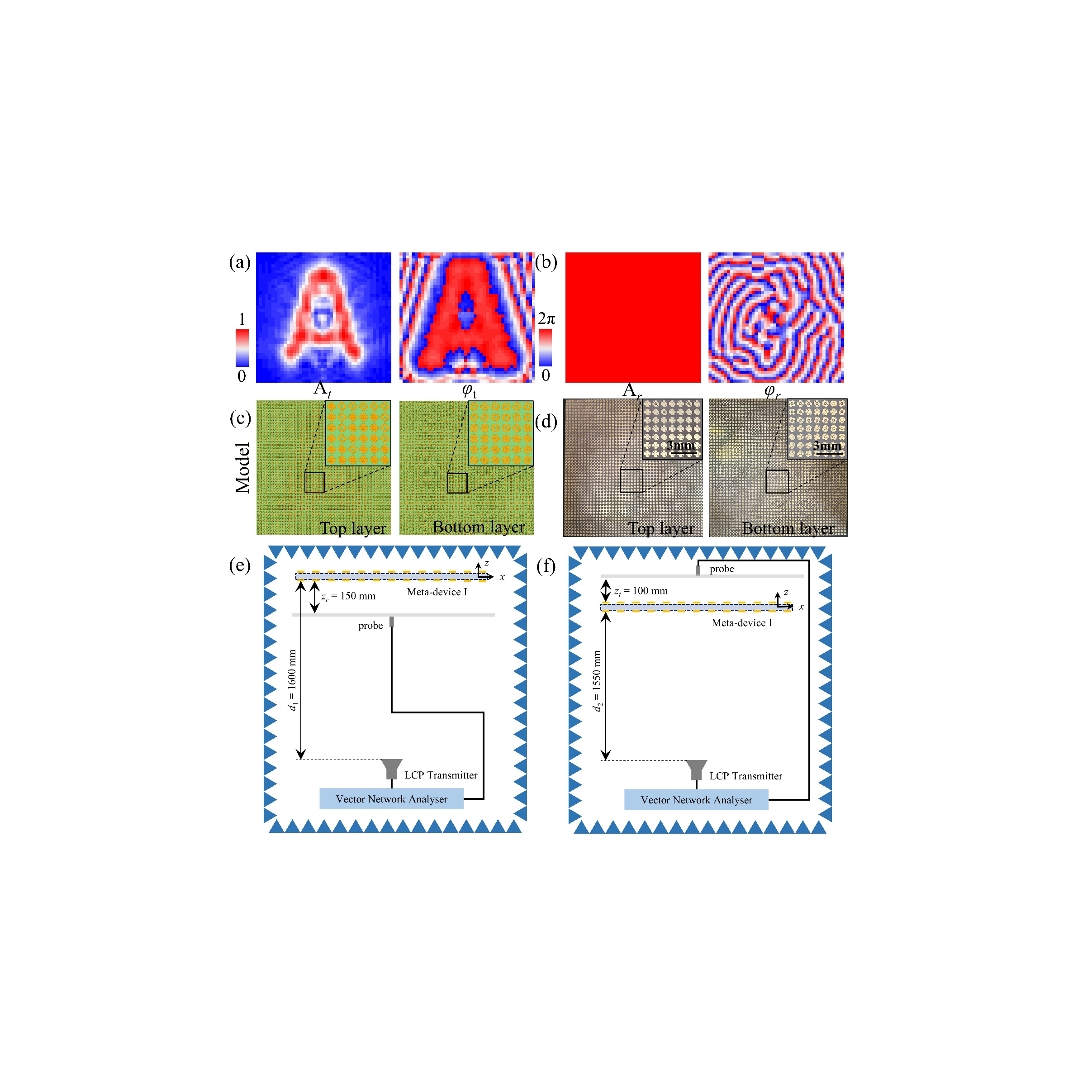


1. Additional results for numerical and experimental characterization of polarization‑routing transmissive-reflective holography. Amplitude and phase response for letter (a) ‘A’ and (b) ‘P’. Top layer and bottom layer of the (c) metasurface model and (d) sample. Schematic diagram of (e) reflection and (f) transmission experiment setups for a dual-channel hologram.

# S7. Polarization‑routing asymmetric beamformer and communication via MWS

To experimentally validate the potential of our polarization-varying metasurface for communication applications, we designed a polarization‑routing asymmetric beamformer and constructed a communication system using a microwave spectrometer (MWS). A photograph of the fabricated sample is shown in Fig. S17b. Its far-field radiation pattern was subsequently measured using the experimental setup illustrated in Fig. S17a. In communication validation, both the MWS transmitter and receiver employ amplitude-modulation circuits for baseband modulation and demodulation, respectively, and communicate through an RS-422 serial communication protocol at a rate of 100 kbps. Detailed hardware architecture is provided in Fig. 5c (i) and (ii).


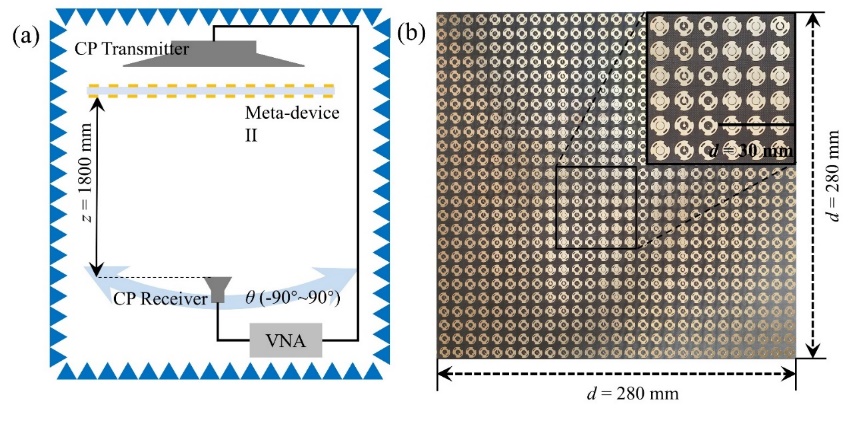


1. Experimental setup for far-field radiation patterns and fabricated sample. (a) Experiment setups. (b) Photograph of the top layer of the fabricated prototype.

For text transmission, text input on the transmitter's touchscreen is digitized and processed by the central processing unit (CPU) for encoding and modulation, generating a digital baseband signal. This signal is converted to an analog form via a digital-to-analog converter (DAC), and then upconverted by a phase-locked carrier to yield the radio frequency (RF) output. After power adjustment by an attenuator, the RF signal is radiated by the LCP horn antenna to illuminate the metadevice, which reshapes the amplitude, phase, and polarization of the EM waves. At the receiver side, the incident RF signal is captured by the RCP horn antenna, then amplified by a low-noise amplifier, and filtered to suppress out-of-band noise. A logarithmic detector monitors signal strength and feeds the data to the CPU for demodulation and error-correction decoding, allowing recovery of the original information. The decoded text is finally rendered on the receiver’s touchscreen.

For audio transmission, audio input is first conditioned by a signal conditioning circuit, then processed by a switch and converted into an analog signal via a digital-to-analog converter (D/A). This analog signal is processed by the CPU (via SPI interface 1) to control the phase-locked source, which generates a carrier signal. After power adjustment through an attenuator, the signal is amplified by an excitation head and transmitted through a transmitting antenna, with further wavefront shaping by the metasurface to manipulate electromagnetic wave properties. At the receiver end, the incident signal is captured by the receiving antenna, then amplified using a low-noise amplifier, and filtered to suppress out-of-band noise. A logarithmic detector converts the signal into a digital form via an analog-to-digital converter (A/D, via SPI interface 2) and feeds it back to the CPU. The CPU performs demodulation to recover the original audio information, which is amplified by an audio power amplifier and finally output through the audio horn.

# S8. Fabrication process for receiver-transmitter metasurface

The fabrication of receive-transmit metasurfaces was carried out using a process involving back-drilling, backfilling, and metallization etching. The fabrication flow-chart is illustrated in Fig. S18a. First, the metallized pattern for each layer was etched and laminated according to the predefined design. Then, via holes were drilled and metalized based on the positions of two metalized vias. Subsequently, excess metallized portions were removed via back-drilling. Next, the vias were backfilled with epoxy resin. Finally, a copper foil was applied to the surface, and electroplating was performed to fill and planarize the structure according to the predetermined pattern. The finalized architecture is depicted in Fig. S19b.


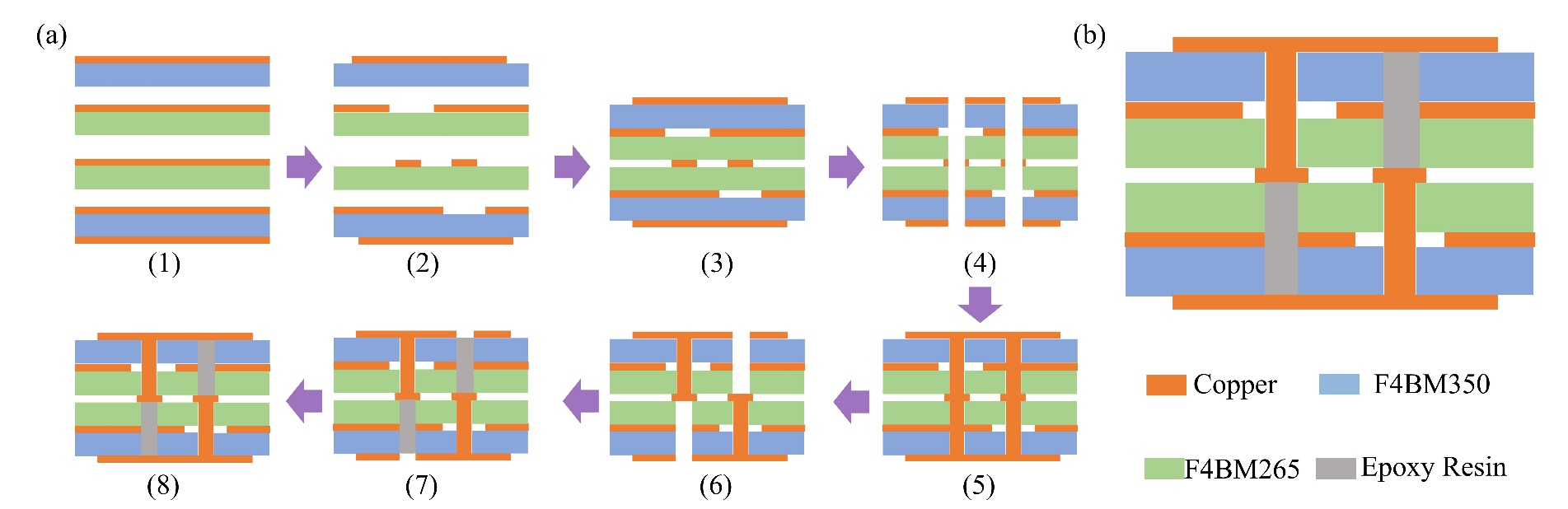


1. Fabrication process for the proposed receiver-transmitter metasurface. (a) flow-chart of the metasurface, comprising: (1-3) Multilayer substrate etching and lamination. (3-5) Via machining and metallization. (5-7) Back-drilling and epoxy resin backfilled. (7-8) Electroplating planarization with copper foil to repair predefined geometric patterns. (b) Final structure achieved through this process.

# Reference

[1] N. Yu, P. Genevet, M.A. Kats. et al.,. Light propagation with phase discontinuities: generalized laws of reflection and refraction. [J]. *Science*, 2011, **334**(6054): 333-337

[2] N.I. Landy, S. Sajuyigbe, J.J. Mock. et al.,. Perfect metamaterial absorber. [J]. *Physical review letters*, 2008, **100**(20): 207402

[3] J.P. Balthasar Mueller, N.A. Rubin, R.C. Devlin. et al.,. Metasurface polarization optics: independent phase control of arbitrary orthogonal states of polarization. [J]. *Physical review letters*, 2017, **118**(11): 113901

[4] Q. Fan, M. Liu, C. Zhang. et al.,. Independent amplitude control of arbitrary orthogonal states of polarization via dielectric metasurfaces. [J]. *Physical review letters*, 2020, **125**(26): 267402

[5] Y. Yuan, S. Sun, Y. Chen. et al.,. A fully phase-modulated metasurface as an energy-controllable circular polarization router. [J]. *Advanced Science*, 2020, **7**(18): 2001437

[6] H. Ren, X. Fang, J. Jang. et al.,. Complex-amplitude metasurface-based orbital angular momentum holography in momentum space. [J]. *Nature Nanotechnology*, 2020, **15**(11): 948-955

[7] Y. Yuan, K. Zhang, Q. Wu. et al.,. Reaching the efficiency limit of arbitrary polarization transformation with non-orthogonal metasurfaces. [J]. *Nature Communications*, 2024, **15**(1): 6682

[8] Y. Bao, Q. Weng, B. Li. Conversion between arbitrary amplitude, phase, and polarization with minimal degrees of freedom of metasurface. [J]. *Laser & Photonics Reviews*, 2022, **16**(2): 2100280
